# Supplementary material for: The impact of a health professional recommendation on weight loss attempts in overweight and obese British adults: a cross-sectional analysis
Source: BMJ Open. 2013 Nov 4;3(11):e003693. doi: 10.1136/bmjopen-2013-003693 (PMC3822310; doi:10.1136/bmjopen-2013-003693)
Supplement: Author's manuscript [file bmjopen-2013-003693.draft_revisions.pdf]

The impact of a health professional recommendation on weight loss attempts in overweight and obese British adults: a cross-sectional analysis

Journal:	*BMJ Open*
Manuscript ID:	bmjopen-2013-003693
Article Type:	Research
Date Submitted by the Author:	31-Jul-2013
Complete List of Authors:	Young, Sarah; University College London, Epidemiology and Public Health<br>Wardle, Jane; University College London, Dept. of Epidemiol<br>Johnson, Fiona; University College London, Dept. of Epidemiol<br>Finer, Nicholas; UCL, Bariatric Medicine and Surgery<br>Beeken, Rebecca; University College London, Dept. of Epidemiol
**Primary Subject Heading**:	General practice / Family practice
Secondary Subject Heading:	Nutrition and metabolism
Keywords:	weight loss, lose weight, health professional advice, doctor advice, PRIMARY CARE

SCHOLARONE™
Manuscripts

For peer review only

STROBE Statement—checklist of items that should be included in reports of observational studies

	Item No	Recommendation	
**Title and abstract**	1	(*a*) Indicate the study's design with a commonly used term in the title or the abstract	Yes
		(*b*) Provide in the abstract an informative and balanced summary of what was done and what was found	Yes
**Introduction**			
Background/rationale	2	Explain the scientific background and rationale for the investigation being reported	Yes
Objectives	3	State specific objectives, including any prespecified hypotheses	Yes
**Methods**			
Study design	4	Present key elements of study design early in the paper	Yes
Setting	5	Describe the setting, locations, and relevant dates, including periods of recruitment, exposure, follow-up, and data collection	Yes
Participants	6	(*a*) *Cohort study*—Give the eligibility criteria, and the sources and methods of selection of participants. Describe methods of follow-up<br>*Case-control study*—Give the eligibility criteria, and the sources and methods of case ascertainment and control selection. Give the rationale for the choice of cases and controls<br>*Cross-sectional study*—Give the eligibility criteria, and the sources and methods of selection of participants	Yes
		(*b*) *Cohort study*—For matched studies, give matching criteria and number of exposed and unexposed<br>*Case-control study*—For matched studies, give matching criteria and the number of controls per case	NA
Variables	7	Clearly define all outcomes, exposures, predictors, potential confounders, and effect modifiers. Give diagnostic criteria, if applicable	Yes
Data sources/ measurement	8*	For each variable of interest, give sources of data and details of methods of assessment (measurement). Describe comparability of assessment methods if there is more than one group	Yes
Bias	9	Describe any efforts to address potential sources of bias	Yes
Study size	10	Explain how the study size was arrived at	Yes
Quantitative variables	11	Explain how quantitative variables were handled in the analyses. If applicable, describe which groupings were chosen and why	Yes
Statistical methods	12	(*a*) Describe all statistical methods, including those used to control for confounding	Yes
		(*b*) Describe any methods used to examine subgroups and interactions	NA
		(*c*) Explain how missing data were addressed	Yes
		(*d*) *Cohort study*—If applicable, explain how loss to follow-up was addressed<br>*Case-control study*—If applicable, explain how matching of cases and controls was addressed<br>*Cross-sectional study*—If applicable, describe analytical methods taking account of sampling strategy	Yes
		(*e*) Describe any sensitivity analyses	NA

Continued on next page

For peer review only

**Results**			
Participants	13*	(a) Report numbers of individuals at each stage of study—eg numbers potentially eligible, examined for eligibility, confirmed eligible, included in the study, completing follow-up, and analysed	Yes
		(b) Give reasons for non-participation at each stage	Yes
		(c) Consider use of a flow diagram	No
Descriptive data	14*	(a) Give characteristics of study participants (eg demographic, clinical, social) and information on exposures and potential confounders	Yes
		(b) Indicate number of participants with missing data for each variable of interest	Yes
		(c) *Cohort study*—Summarise follow-up time (eg, average and total amount)	NA
Outcome data	15*	*Cohort study*—Report numbers of outcome events or summary measures over time	NA
		Case-control study—Report numbers in each exposure category, or summary measures of exposure	NA
		Cross-sectional study—Report numbers of outcome events or summary measures	Yes
Main results	16	(*a*) Give unadjusted estimates and, if applicable, confounder-adjusted estimates and their precision (eg, 95% confidence interval). Make clear which confounders were adjusted for and why they were included	Yes
		(*b*) Report category boundaries when continuous variables were categorized	NA
		(*c*) If relevant, consider translating estimates of relative risk into absolute risk for a meaningful time period	NA
Other analyses	17	Report other analyses done—eg analyses of subgroups and interactions, and sensitivity analyses	NA
**Discussion**			
Key results	18	Summarise key results with reference to study objectives	Yes
Limitations	19	Discuss limitations of the study, taking into account sources of potential bias or imprecision. Discuss both direction and magnitude of any potential bias	Yes
Interpretation	20	Give a cautious overall interpretation of results considering objectives, limitations, multiplicity of analyses, results from similar studies, and other relevant evidence	Yes
Generalisability	21	Discuss the generalisability (external validity) of the study results	Yes
**Other information**			
Funding	22	Give the source of funding and the role of the funders for the present study and, if applicable, for the original study on which the present article is based	Yes

*Give information separately for cases and controls in case-control studies and, if applicable, for exposed and unexposed groups in cohort and cross-sectional studies.

Note: An Explanation and Elaboration article discusses each checklist item and gives methodological background and published examples of transparent reporting. The STROBE checklist is best used in conjunction with this article (freely available on the Web sites of PLoS Medicine at http://www.plosmedicine.org/, Annals of Internal Medicine at http://www.annals.org/, and Epidemiology at http://www.epidem.com/). Information on the STROBE Initiative is available at www.strobe-statement.org.

For peer review only

For peer review only - http://bmjopen.bmj.com/site/about/guidelines.xhtml

The impact of a health professional recommendation on weight loss attempts in overweight and obese British adults: a cross-sectional analysis

Sarah E Young, Jane Wardle, Fiona Johnson, Nicholas Finer, Rebecca J Beeken

Department of Epidemiology and Public Health, University College London, London WC1E 6BT, UK

Sarah E Young

PhD student

Jane Wardle

Professor of Clinical Psychology

Fiona Johnson

Research Psychologist

Rebecca J Beeken

Senior Research Psychologist

UCL Institute of Cardiovascular Science, 170 Tottenham Court Road, London W1T 7HA, UK

Nicholas Finer

Honorary Professor

Correspondence to: RJ Beeken. Email: r.beeken@ucl.ac.uk. Telephone: +44 (0)20 7679 1720.

Keywords weight loss; lose weight; health professional advice; doctor advice; primary care.

Word count 2,377

For peer review only

ABSTRACT

Objectives To examine the effect that health professional (HP) advice to lose weight has on overweight and obese adults' motivation to lose weight and attempts to lose weight.

Design Cross-sectional survey.

Setting Great Britain.

Participants 810 overweight or obese (BMI ≥25kg/m^2) adults.

Main outcome measures Participants were asked if they had ever received HP advice to lose weight and reported their desire to weigh less (preferred weight ≤95% of current weight) and whether they were attempting to lose weight.

Results Only 17% of overweight and 42% of obese respondents recalled ever having received HP advice to lose weight. HP advice was associated with wanting to weigh less (89% vs. 61% among those not receiving advice) and attempting to lose weight (68% vs. 37%). In multivariable analyses, HP advice to lose weight was associated with increased odds of wanting to weigh less (odds ratio (OR) = 3.71, 95% CI = 2.10–6.55) and attempting to lose weight (OR = 3.53, 95% CI = 2.44–5.10) independent of demographic characteristics and weight status.

Conclusions HP advice to lose weight appears to increase motivation to lose weight and weight loss behaviour, but only a minority of overweight or obese adults receive such advice. Better training for HPs in delivering brief weight counselling could offer an opportunity to improve obese patients' motivation to lose weight.

For peer review only

ARTICLE SUMMARY

Article focus

- Health professionals are recommended to routinely speak to overweight and obese patients about their weight and offer weight control advice, but many doubt the effectiveness of providing advice.
- This study examined associations between health professional advice for weight loss and patients' motivation to lose weight in a British sample of overweight and obese adults.

Key messages

- Overweight and obese individuals who have received health professional advice to lose weight are over three times as likely to attempt weight loss as those who have never received advice.

Strengths and limitations

- This is the first study to show an association between health professional advice to lose weight and weight loss attempts in a British sample.
- Data were not collected on actual weight loss so the impact of health professional advice on weight loss outcomes could not be established.

For peer review only - http://bmjopen.bmj.com/site/about/guidelines.xhtml

INTRODUCTION

In most Western countries, between 40% and 70% of adults have a body weight that places them at risk of developing weight-related long-term health problems.[1,2] There is good evidence that losing as little as 5% of body weight confers significant cardio-metabolic benefit for overweight and obese individuals,3,4 although sustained weight loss is difficult to achieve.[5,6]

The majority of overweight and obese adults say they want to lose weight,[7] but only around half report actively trying to lose weight.[7,8] One potential source of motivation to lose weight is advice from a health professional. Physicians and other health professionals have a unique opportunity to raise obese patients' awareness of their weight status and its associated health risks, and to offer advice on weight management. In the US, doctors are recommended to screen for obesity and refer all obese patients for weight loss treatment.[9] Recent reports by the Academy of Medical Royal Colleges (AMRC) and the Royal College of Physicians (RCP) have called for similar action in the UK.[10,11] Under the policy of 'making every contact count', the AMRC recommended that health professionals should routinely speak to overweight and obese patients about diet and exercise habits at each appointment and offer help. This recommendation is also made directly to health professionals in the UK via NICE and SIGN guidelines, and through the quality and outcomes framework (QOF).[12–14]

In spite of these recommendations, current figures indicate that only a minority of health professionals routinely give weight advice.[15–20] A range of barriers, including perceived lack of time, inadequate knowledge, lack of training or confidence and inadequate teaching materials have been identified as contributing to the relatively low rates of weight control advice from health professionals.[21–27] Importantly, many health professionals also doubt the usefulness of providing weight advice because they don't feel it will change patients' behaviour.[21,23,26,28]

However, there is evidence suggesting that health professionals can play a valuable role in helping patients to change their behaviour. Advice and involvement of health professionals in helping smokers quit is effective, and has led to evidence-based guidelines for their active intervention.[29] A recent systematic review and meta-analysis of US studies concluded that weight loss advice from a health professional in primary care is associated with positive weight loss behaviour change in overweight and obese patients,[30] but no equivalent studies have been conducted in the UK population.

For peer review only - http://bmjopen.bmj.com/site/about/guidelines.xhtml

For peer review only

This study therefore tested the hypothesis that advice to lose weight from a doctor or other health professional would be associated with a greater likelihood of trying to lose weight in a UK sample of overweight and obese adults.

METHODS

Design and participants

Data on weight loss motivation and weight loss advice were collected from a large sample of British adults (n=1,986, 932 men, 1,054 women) as part of a home-based, face-to-face survey from across Great Britain in April 2012. To reduce potential bias, data were collected by an independent market research company (TNS) that had no knowledge of our study aims. TNS uses a random location methodology based on the 2001 Census small-area statistics and the postcode address file, stratified by Government Office Region and social grade, to select sample points. At each location, quotas are set for age, gender, children in the home, and working status to ensure a balanced sample of adults within effective contacted addresses. Interviewers were instructed to leave three doors between each successful interview.

This survey was designed as part of an ongoing study assessing changes in weight perceptions in the British population (see Johnson et al. [31] for findings of the previous two surveys in this series), but it seemed timely given recent recommendations to also use the data collected to explore the relationship between health professional advice to lose weight and weight loss motivation. The majority of respondents provided height and weight data (n=1,557). These analyses focus on respondents with a BMI in the overweight or obese range (BMI ≥25; n=810).

Measures

Weight loss motivation: Respondents were asked how much they would ideally like to weigh, with desire to weigh less indexed by reporting an ideal weight ≤95% of self-reported current weight (i.e. ≥5% weight loss). They were also asked which of the following statements best described them: 1) *I'm not bothered about my weight*; 2) *I watch my weight to keep it where it is now*; 3) *I'm trying to lose weight*; or 4) *I'm trying to gain weight*. Respondents indicating they were trying to lose weight were compared with the other groups combined.

Health professional advice to lose weight: Respondents were asked whether a doctor or other health professional had ever told them that they should lose weight (yes/no).

For peer review only - http://bmjopen.bmj.com/site/about/guidelines.xhtml

Current anthropometry: Height and weight were self-reported in metric or imperial units as the respondent preferred. BMI (kg/m^2) was calculated from self-reported heights and weights.

Demographics: Age, sex, ethnicity, marital status, and socioeconomic status (SES) were included in the analyses. Due to the small number of participants from any individual ethnic minority group, ethnicity was only categorised as white vs. non-white. Marital status was categorised as married/living as married vs. unmarried. SES was defined according to the National Readership Survey classification of the person's current or last occupation,[32] and dichotomised into ABC1 (higher SES) vs. C2DE (lower SES).

Statistical analyses

Analyses were done using IBM SPSS Statistics version 19, with weighted data to match the population characteristics. Variables used for weighting included age, sex, social grade, and standard region. Analyses were repeated on unweighted data with no significant differences in the results, so with the exception of the sample description, only analyses on weighted data are reported.

Descriptive statistics (unweighted data) are presented for sample characteristics and prevalence of weight loss motivation and weight loss recommendations. Multivariable logistic regression (weighted data) was used to examine associations between health professional advice to lose weight and weight loss motivation controlling for age, sex, ethnicity, marital status, SES, and weight status.

There was very little missing data (0.4% for health professional advice, 9.4% for desire to weigh less, 1.7% for attempting to lose weight, 0% for demographic variables). Analyses were run only for participants who had provided full data on the relevant variables.

RESULTS

Weight and height data were provided by 1,557 out of 1,986 respondents, of whom 48 (3%) had a BMI within the underweight range (BMI <18.5), 699 (45%) were in the healthy weight range, 528 (34%) were overweight (BMI ≥25 and <30), and a further 282 (18%) were obese (BMI ≥30). Subsequent analyses are restricted to respondents whose BMI defined them as overweight or obese (n=810).

Demographic and anthropometric characteristics of the overweight and obese respondents are shown in Table 1. Their mean age was 51.3 years (range: 16 to 90 years), 47% were female, 89%

For peer review only - http://bmjopen.bmj.com/site/about/guidelines.xhtml

For peer review only

were white, 62% were married or living as married, and 61% were lower SES. Their mean weight was 85.3kg and mean BMI was 29.6kg/m^2.

Overall, a quarter (26%) of the overweight or obese respondents (17% of overweight and 42% of obese) reported ever having received health professional advice to lose weight. Mean BMI was higher among those who had received health professional advice to lose weight (32.1kg/m^2, SD 5.2) than had not (28.7kg/m^2, SD 3.5; $t(276.93) = 8.60$, $P<0.001$). Health professional advice appeared to become normative (i.e. was reported by more than 50% of respondents) at a BMI of approximately 37kg/m^2 (see Figure 1).

Overall, 68% of respondents wanted to weigh less, and 45% were currently trying to lose weight. Receiving health professional advice to lose weight was associated with higher prevalence of wanting to weigh less (89% vs. 61%) and of attempting weight loss (68% vs. 37%). This pattern was the same for both obese and overweight participants (see Figure 2).

The results of the multivariable logistic regression models are shown in Table 2. Compared with overweight respondents, obese respondents were more likely to want to lose weight (OR=8.57) and also more likely to be attempting to lose weight (OR=1.91). Being female quadrupled the odds of wanting to weigh less (OR=4.39) and doubled the odds of weight loss attempts (OR=1.93). The odds of attempting weight loss were significantly lower in older respondents (OR=0.52) and higher in higher SES respondents (OR=1.40), but there was no significant association with marital status or ethnicity. After controlling for the effects of demographics and weight status, having received health professional advice to lose weight more than tripled the odds of both wanting to weigh less (OR=3.71) and trying to lose weight (OR=3.53).

DISCUSSION

In this population-based sample of overweight and obese British adults, only a quarter (26%) reported having received advice to lose weight from a health professional. Around two-thirds reported a desire to weigh at least 5% less than their current weight, and just under half said they were actively trying to lose weight. Having received health professional advice to lose weight was strongly associated with both wanting to weigh less and trying to lose weight after controlling for demographic characteristics.

Healthcare providers in primary care are required to record BMI on all patients, and recommended to discuss diet and exercise with all overweight and obese patients.[12–14] However, in this sample,

For peer review only - http://bmjopen.bmj.com/site/about/guidelines.xhtml

For peer review only

fewer than half (42%) of obese respondents and only 17% of overweight respondents reported having been recommended by a doctor or other health professional to lose weight; findings similar to US studies.[15–20] Nonetheless, this is considerably higher than the 5% (among overweight respondents) and 16% (among obese respondents) reported in a similar survey in 2001.[8] Health professionals report an array of barriers to providing weight loss advice, including perceived lack of time, knowledge, training, and confidence.[21–27] Perhaps most importantly, obesity treatment is often perceived by physicians to be a daunting or even futile task,[28] and many say they find it professionally unrewarding.[33–37] This could well affect their enthusiasm to broach the topic.

Despite the notorious difficulty in achieving and maintaining significant weight loss, one study found that almost half of overweight and obese individuals believed they could lose weight if they felt they needed to.[23] This may highlight an important role for health professionals to give clear advice to patients when weight loss is needed. Our results demonstrate that receiving health professional advice to lose weight was associated with desiring a lower body weight, and more importantly, with attempting weight loss. Odds ratios were similar to those observed in a meta-analysis of US studies.[30] Among respondents who desired to weigh less, health professional advice was associated with being significantly more likely to be trying to lose weight. The translation of behavioural intentions to changes in behaviour is known to be a major block in lifestyle interventions.[38] The finding observed here suggests that in the case of weight loss, advice from a health professional can help to bridge the intention-behaviour gap.

Together, these results provide strong support for the recommendation that physicians and other health professionals should discuss weight with overweight and obese patients.[9,10] Targeted education and training programmes on weight counselling for health professionals could help overcome some of the barriers that hold them back from 'making every contact count'.[10]

The findings of this study are subject to several limitations. We do not have response rate information because of the method of sampling, but as the primary focus is the association between weight loss advice and weight loss attempts, the sampling method is not likely to influence the findings. However, in terms of prevalence of weight loss advice and weight loss attempts, the results only reflect the experience of individuals who agreed to take part; which may overestimate levels of weight concern. Use of self-reported weights and heights mean that BMI is likely to be underestimated.[39,40] Consistent with this, the prevalence of obesity was lower than figures based on measured anthropometric data from the most recent Health Survey for England [41] (18% in the present study vs. 26% in the HSE). This may limit the extent to which findings can be generalised to the entire overweight and obese population.

For peer review only - http://bmjopen.bmj.com/site/about/guidelines.xhtml

The finding that only a minority of overweight and obese people had been advised to lose weight was important, but it is possible that more had received advice than recalled it. Health professional-reported rates of weight counselling are markedly higher than the 40-50% reported by obese patients,[42] although there is some evidence to suggest health professionals may overestimate the level of intervention they provide to patients.[42] Guiding health professionals on how to provide weight control advice in a way that resonates more strongly with their patients could improve the effectiveness of the advice and reduce any reservations about its utility.

These data do not address the reasons for being given or not given weight loss advice. People who had not received advice may not have seen a health professional since they became overweight or obese, they may have seen a health professional who did not identify their weight status, or the health professional may have recognised their overweight/obesity but had reasons not to mention it. Identifying the determinants of giving weight loss advice could further help tailor health professional training.

The use of a cross-sectional design meant we were not able to determine whether health professional advice increased motivation to lose weight on an individual level. Individuals who are already concerned about their health may visit their doctors more often, and therefore have more opportunities to elicit weight loss advice, and they may even have asked for advice directly. The design also precluded assessment of weight loss success following health professional advice, although previous research has demonstrated better long-term weight outcomes in treatment programmes following a medical trigger for weight loss,[30] and extrapolation from studies of GP advice for smoking cessation [29] give some cause for optimism. Prospective longitudinal research is needed to provide insight into motivational changes and actual weight reduction following advice to lose weight.

The results of this study confirm that many overweight and obese adults in the UK express a desire to weigh less than they do, but notably fewer are actively trying to lose weight. Advice from a health professional was strongly associated with attempting weight loss; supporting the recommendation that health professionals should discuss weight with overweight and obese patients as a matter of routine. Better training for health professionals in discussing weight issues could make a significant contribution to population weight management.

For peer review only

TABLES

Table 1 Sample demographic and anthropometric characteristics among overweight and obese respondents (n=810). Values are numbers (percentages) unless stated otherwise

**Demographic and healthcare characteristics**	
Age (years)	
Mean (SD)	51.3 (17.9)
<55	447 (55.2)
≥55	363 (44.8)
Sex	
Male	432 (53.5)
Female	378 (46.7)
Ethnicity	
White	721 (89.0)
Non-white	88 (10.9)
Marital status	
Unmarried	312 (38.5)
Married/living as married	498 (61.5)
SES	
Lower	493 (60.9)
Higher	317 (39.1)
Health professional advice to lose weight	
No	599 (74.0)
Yes	208 (25.7)
**Anthropometric characteristics**	
Mean (SD) height, cm	169.4 (10.7)
Mean (SD) weight, kg	85.3 (16.2)
Mean (SD) BMI, kg/m^2	29.6 (4.3)
Weight status	
Overweight	528 (65.2)
Obese	282 (34.8)

SES = socio-economic status. Note: Numbers may not sum to total sample number, as some items were not answered by all participants. Percentages were derived from the full sample and may therefore not sum to 100%.

For peer review only

For peer review only - http://bmjopen.bmj.com/site/about/guidelines.xhtml

Table 2 Multivariable logistic regression models predicting desire to weigh less and attempting to lose weight in overweight and obese UK adults (weighted data)

	Desire to weigh less			Attempting to lose weight		
**Characteristics**	**OR**	**95% CI**	***P***	**OR**	**95% CI**	***P***
Age (years)						
<55	1.00	-	-	1.00	-	-
≥55	0.84	0.57-1.23	0.369	0.52	0.38-0.71	<0.001
Sex						
Male	1.00	-	-	1.00	-	-
Female	4.39	2.94-6.56	<0.001	1.93	1.43-2.63	<0.001
Ethnicity						
White	1.00	-	-	1.00	-	-
Non-white	0.96	0.51-1.84	0.912	1.11	0.67-1.85	0.686
Marital status						
Unmarried	1.00	-	-	1.00	-	-
Married/living as married	0.87	0.59-1.29	0.483	1.34	0.98-1.84	0.065
SES						
Lower	1.00	-	-	1.00	-	-
Higher	1.34	0.92-1.95	0.125	1.40	1.03-1.89	0.030
Weight status						
Overweight	1.00	-	-	1.00	-	-
Obese	8.57	4.87-15.08	<0.001	1.91	1.38-2.64	<0.001
Health professional advice to lose weight						
No	1.00	-	-	1.00	-	-
Yes	3.71	2.10-6.55	<0.001	3.53	2.44-5.10	<0.001

SES = socio-economic status; OR = odds ratio; CI = confidence interval.

For peer review only

For peer review only - http://bmjopen.bmj.com/site/about/guidelines.xhtml

FIGURES

Figure 1 Percentage reporting health professional advice to lose weight by BMI

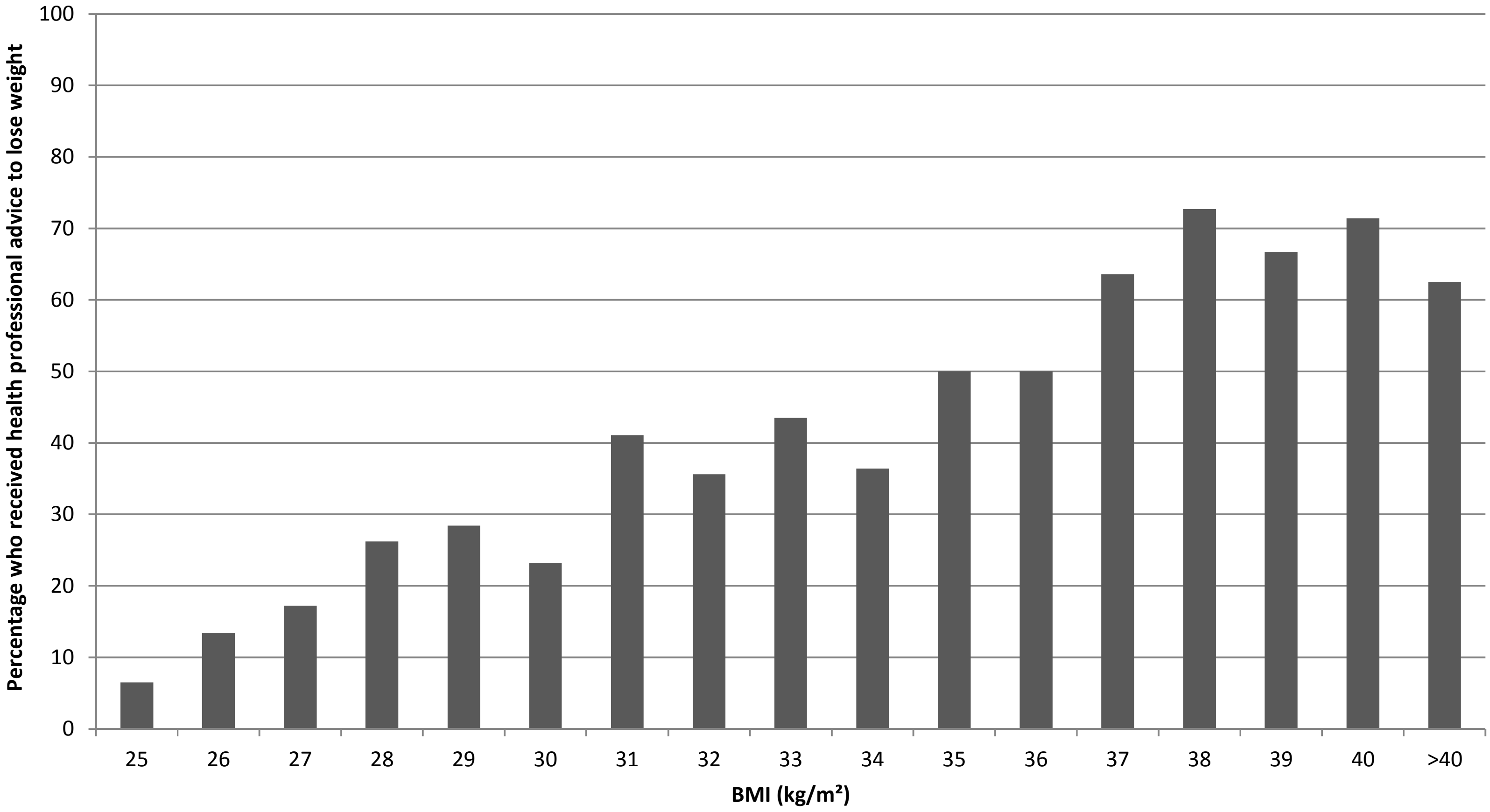

Note: BMI points were rounded down such that a BMI point of 25 includes 25.0-25.9.

For peer review only - http://bmjopen.bmj.com/site/about/guidelines.xhtml

Figure 2 Prevalence of attempting to lose weight by health professional advice to lose weight and weight status

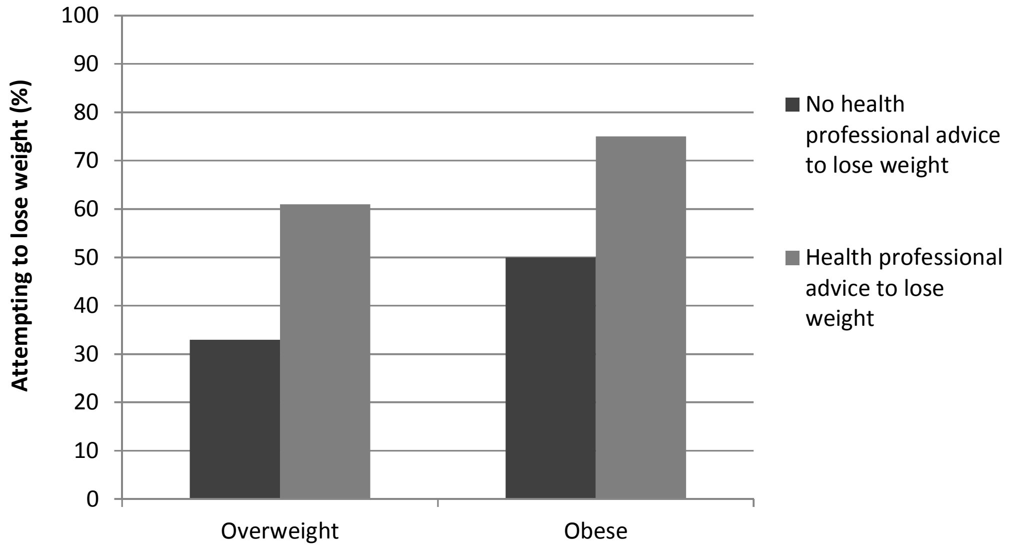

For peer review only - http://bmjopen.bmj.com/site/about/guidelines.xhtml

CONTRIBUTORSHIP STATEMENT

Everyone listed as an author fulfils all three of the ICMJE guidelines for authorship: 1) substantial contributions to conception and design, acquisition of data, or analysis and interpretation of data; 2) drafting the article or revising it critically for important intellectual content; and 3) final approval of the version to be published.

SEY, JW, FJ, NF, and RJB were responsible for the study concept and design. JW obtained funding. JW and FJ acquired the data. SEY, JW, FJ, NF, and RJB analysed and interpreted the data. SEY did the statistical analysis. SEY drafted the manuscript, and all authors revised it for important intellectual content. All authors had final approval of the version to be published. JW is the guarantor.

All authors had full access to all of the data (including statistical reports and tables) in the study and can take responsibility for the integrity of the data and the accuracy of the data analysis.

COMPETING INTERESTS

All authors have completed the Unified Competing Interest form at www.icmje.org/coi_disclosure.pdf (available on request from the corresponding author) and declare: support for the study by grants from the UK Medical Research Council, UK Economic and Social Research Council, and Cancer Research UK; no financial relationships with any organisations that might have an interest in the submitted work in the previous three years; and no other relationships or activities that could appear to have influenced the submitted work.

FUNDING

This study was supported by grants from the UK Medical Research Council, UK Economic and Social Research Council, and Cancer Research UK.

The study sponsors had no role in the study design; in the collection, analysis, and interpretation of the data; in the preparation, review, or approval of the manuscript; or in the decision to submit it for publication. The researchers were independent of the funding agencies.

For peer review only

For peer review only - http://bmjopen.bmj.com/site/about/guidelines.xhtml

REFERENCES

1. Guh DP, Zhang W, Bansback N, Amarsi Z, Laird Birmingham C, Anis AH. The incidence of co-morbidities related to obesity and overweight: A systematic review and meta-analysis. BMC Public Health 2009;**9**:88 (2009).
2. Ford ES, Mokdad AH. Epidemiology of obesity in the western hemisphere. JCEM 2008;**93**:S1–S8.
3. Blackburn, G. Effect of degree of weight loss on health benefits. Obes Res 1995;**3**:S211–S216.
4. Lavie CJ, Milani RV, Artham SM, Patel D, Ventura HO. The obesity paradox, weight loss, and coronary disease. Am J Med 2009;**122**:1106–1114.
5. Franz MJ, VanWormer JJ, Crain AL, Boucher JL, Histon T, Caplan W et al. Weight-loss outcomes: a systematic review and meta-analysis of weight-loss clinical trials with a minimum 1-year follow-up. J Am Diet Ass 2007;**107**:1755–1767.
6. Tsai AG, Wadden TA. Systematic review: an evaluation of major commercial weight loss programs in the United States. Ann Intern Med 2005;**142**:56–66.
7. Yaemsiri S, Slining MM, Agarwal SK. Perceived weight status, overweight diagnosis, and weight control among US adults: the NHANES 2003–2008 Study. Int J Obes 2011;**35**:1063–1070.
8. Wardle J, Johnson F. Weight and dieting: examining levels of weight concern in British adults. Int J Obes 2002;**26**:1144–1149.
9. Moyer VA. Screening for and management of obesity in adults: U.S. Preventive Services Task Force recommendation statement. Ann Intern Med 2012;**157**:373–378 (2012).
10. Academy of Medical Royal Colleges. Measuring Up: The medical profession's prescription for the nation's obesity crisis. Academy of Medical Royal Colleges, 2013. http://www.aomrc.org.uk/projects/obesity-steering-group.html (accessed 17 Jun 2013).
11. Royal College of Physicians. The training of health professionals for the prevention and treatment of overweight and obesity. Royal College of Physicians, 2010. http://www.rcplondon.ac.uk/sites/default/files/press_releases/2010/12/obesity-report-2010.pdf (accessed 17 Jun 2013).
12. NHS Employers. 2013/14 general medical services (GMS) contract quality and outcomes framework (QOF). 2013. http://www.nhsemployers.org/Aboutus/Publications/Documents/qof-2013-14.pdf (accessed 17 Jun 2013).
13. National Institute for Health and Clinical Excellence (NICE). Obesity: the prevention, identification, assessment and management of overweight and obesity in adults and children. NICE, 2006. http://www.nice.org.uk/ (accessed 17 Jun 2013).
14. Scottish Intercollegiate Guidelines Network (SIGN). Management of Obesity: A national clinical guideline. SIGN, 2010. http://www.sign.ac.uk/guidelines/fulltext/115/ (accessed 17 Jun 2013).
15. Clune A, Fischer JG, Lee JS, Reddy S, Johnson MA, Hausman DB. Prevalence and predictors of recommendations to lose weight in overweight and obese older adults in Georgia senior centers. Prev Med 2010;**51**:27–30.

For peer review only - http://bmjopen.bmj.com/site/about/guidelines.xhtml

16. Galuska DA, Will JC, Serdula MK, Ford ES. Are health care professionals advising obese patients to lose weight? JAMA 1999;**282**:1576–1578.
17. Jackson JE, Doescher MP, Saver BG, Hart LG. Trends in professional advice to lose weight among obese adults, 1994 to 2000. J Gen Intern Med 2005;**20**:814–818.
18. Loureiro ML, Nayga Jr RM. Obesity, weight loss, and physician's advice. Soc Sci Med 2006;**62**:2458–2468.
19. Sciamanna C, Tate D, Lang W, Wing RR. Who reports receiving advice to lose weight?: Results from a multistate survey. Arch Intern Med 2000;**160**:2334–2339.
20. Stafford RS, Farhat JH, Misra B, Schoenfeld DA. National patterns of physician activities related to obesity management. Arch Fam Med 2000;**9**:631–638.
21. Hiddink GJ, Hautvast JG, Van Woerkum CM, Fieren CJ, Van 't Hof MA. Nutrition guidance by primary-care physicians: perceived barriers and low involvement. Eur J Clin Nutr 1995;**49**:842–851.
22. Harris JE, Hamaday V, Mochan E. Osteopathic family physicians' attitudes, knowledge, and self-reported practices regarding obesity. J Am Osteopath Assoc 1999;**99**:358–365.
23. Ruelaz AR, Diefenbach P, Simon B, Lanto A, Arterburn D, Shekelle PG. Perceived barriers to weight management in primary care--perspectives of patients and providers. J Gen Intern Med 2007;**22**:518–522.
24. Kenner MM, Taylor ML, Dunn PC, Gruchow HW, Kolasa K. primary care providers need a variety of nutrition and wellness patient education materials. J Am Diet Assoc 1999;**99**:462–466.
25. Leverence RR, Williams RL, Sussman A, Crabtree BF. Obesity counseling and guidelines in primary care: a qualitative study. Am J Prev Med 2007;**32**:334–339.
26. Foster GD, Wadden TA, Makris AP, Davidson D, Sanderson RS, Allison DB et al. Primary care physicians' attitudes about obesity and its treatment. Obes Res 2003;**11**:1168–1177.
27. Michie S. Talking to primary care patients about weight: A study of GPs and practice nurses in the UK. Psychol Health Med 2007;**12**:521–525.
28. Frank A. Futility and avoidance. Medical professionals in the treatment of obesity. JAMA 1993;**269**:2132–2133.
29. West R, McNeill A, Raw M. Smoking cessation guidelines for health professionals: an update. Thorax 2000;**55**:987–999.
30. Rose SA, Poynter PS, Anderson JW, Noar SM, Conigliaro J. Physician weight loss advice and patient weight loss behavior change: a literature review and meta-analysis of survey data. Int J Obes 2013;**37**:118-128.
31. Johnson F, Cooke L, Croker H, Wardle J. Changing perceptions of weight in Great Britain: comparison of two population surveys. BMJ 2008;**337**:a494–a494.
32. National Readership Survey. Social grade - definitions and discriminatory power. National Readership Survey, 2007. http://www.nrs.co.uk/lifestyle.html (accessed 17 Jun 2013).
33. Bocquier A, Verger P, Basdevant A, Andreotti G, Baretge J, Villani P et al. Overweight and obesity: knowledge, attitudes, and practices of general practitioners in France. Obes Res 2005;**13**:787–795.
34. Campbell K, Crawford D. Management of obesity: attitudes and practices of Australian dietitians. Int J Obes Relat Metab Disord 2000;**24**:701–710.
35. Campbell K, Engel H, Timperio A, Cooper C, Crawford D. Obesity management: Australian general practitioners' attitudes and practices. Obes Res 2000;**8**:459–466.

For peer review only - http://bmjopen.bmj.com/site/about/guidelines.xhtml

36. Loomis GA, Connolly KP, Clinch CR, Djuric DA. Attitudes and practices of military family physicians regarding obesity. Mil Med 2001;**166**:121–125.

37. Thuan JF, Avignon A. Obesity management: attitudes and practices of French general practitioners in a region of France. Int J Obes 2005;**29**:1100–1106.

38. Webb TL, Sheeran P. Does changing behavioral intentions engender behavior change? A meta-analysis of the experimental evidence. Psychol Bull 2006;**132**:249–268.

39. Cameron R, Evers SE. Self-report issues in obesity and weight management: State of the art and future directions. Behav Assess 1990. http://psycnet.apa.org/psycinfo/1990-18712-001 (accessed 17 Jun 2013).

40. Roberts RJ. Can self-reported data accurately describe the prevalence of overweight? Public Health 1995;**109**:275–284.

41. Health Survey for England. Health Survey for England - 2010: Trend tables. Health Survey for England, 2010. http://www.ic.nhs.uk/statistics-and-data-collections/health-and-lifestyles-related-surveys/health-survey-for-england/health-survey-for-england--2010-trend-tables (accessed 17 Jun 2013).

42. Laws R. Current approaches to obesity management in UK Primary Care: the Counterweight Programme. J Hum Nutr Diet 2004;**17**:183–190.

For peer review only

For peer review only - http://bmjopen.bmj.com/site/about/guidelines.xhtml

The impact of a health professional recommendation on weight loss attempts in overweight and obese British adults: a cross-sectional analysis

Journal:	*BMJ Open*
Manuscript ID:	bmjopen-2013-003693.R1
Article Type:	Research
Date Submitted by the Author:	25-Sep-2013
Complete List of Authors:	Jackson, Sarah; University College London, Epidemiology and Public Health<br>Wardle, Jane; University College London, Dept. of Epidemiol<br>Johnson, Fiona; University College London, Dept. of Epidemiol<br>Finer, Nicholas; UCL, Bariatric Medicine and Surgery<br>Beeken, Rebecca; University College London, Dept. of Epidemiol
**Primary Subject Heading**:	General practice / Family practice
Secondary Subject Heading:	Nutrition and metabolism
Keywords:	weight loss, lose weight, health professional advice, doctor advice, PRIMARY CARE

SCHOLARONE™ Manuscripts

For peer review only

The impact of a health professional recommendation on weight loss attempts in overweight and obese British adults: a cross-sectional analysis

Sarah E Jackson, Jane Wardle, Fiona Johnson, Nicholas Finer, Rebecca J Beeken

Department of Epidemiology and Public Health, University College London, London WC1E 6BT, UK

Sarah E Jackson

PhD student

Jane Wardle

Professor of Clinical Psychology

Fiona Johnson

Research Psychologist

Rebecca J Beeken

Senior Research Psychologist

UCL Institute of Cardiovascular Science, 170 Tottenham Court Road, London W1T 7HA, UK

Nicholas Finer

Honorary Professor

Correspondence to: RJ Beeken. Email: r.beeken@ucl.ac.uk. Telephone: +44 (0)20 7679 1720.

Keywords weight loss; lose weight; health professional advice; doctor advice; primary care.

Word count 2,856

For peer review only

ABSTRACT

Objectives To examine the effect that health professional (HP) advice to lose weight has on overweight and obese adults' motivation to lose weight and attempts to lose weight.

Design Cross-sectional survey.

Setting Great Britain.

Participants 810 overweight or obese (BMI ≥25kg/m^2) adults.

Main outcome measures Participants were asked if they had ever received HP advice to lose weight and reported their desire to weigh less (ideal weight ≤95% of current weight) and whether they were attempting to lose weight.

Results Only 17% of overweight and 42% of obese respondents recalled ever having received HP advice to lose weight. HP advice was associated with wanting to weigh less (89% vs. 61% among those not receiving advice) and attempting to lose weight (68% vs. 37%). In multivariable analyses, HP advice to lose weight was associated with increased odds of wanting to weigh less (odds ratio (OR) = 3.71, 95% CI = 2.10–6.55) and attempting to lose weight (OR = 3.53, 95% CI = 2.44–5.10) independent of demographic characteristics and weight status.

Conclusions HP advice to lose weight appears to increase motivation to lose weight and weight loss behaviour, but only a minority of overweight or obese adults receive such advice. Better training for HPs in delivering brief weight counselling could offer an opportunity to improve obese patients' motivation to lose weight.

For peer review only - http://bmjopen.bmj.com/site/about/guidelines.xhtml

ARTICLE SUMMARY

Article focus

- Health professionals are recommended to routinely speak to overweight and obese patients about their weight and offer weight control advice, but many doubt the effectiveness of providing advice.
- This study examined associations between health professional advice for weight loss and patients' motivation to lose weight in a British sample of overweight and obese adults.

Key messages

- Overweight and obese individuals who have received health professional advice to lose weight are over three times as likely to attempt weight loss as those who have never received advice.

Strengths and limitations

- This is the first study to show an association between health professional advice to lose weight and weight loss attempts in a British sample.
- Data were not collected on actual weight loss so the impact of health professional advice on weight loss outcomes could not be established.

For peer review only

For peer review only - http://bmjopen.bmj.com/site/about/guidelines.xhtml

INTRODUCTION

In most Western countries, between 40% and 70% of adults have a body weight that places them at risk of developing weight-related long-term health problems.[1,2] There is good evidence that losing as little as 5% of body weight confers significant cardio-metabolic benefit for overweight and obese individuals,[3,4] although sustained weight loss is difficult to achieve.[5,6]

The majority of overweight and obese adults say they want to lose weight,[7] but only around half report actively trying to lose weight.[7,8] One potential source of motivation to lose weight is advice from a health professional. Physicians and other health professionals have a unique opportunity to raise obese patients' awareness of their weight status and its associated health risks, and to offer advice on weight management. In the US, doctors are recommended to screen for obesity and refer all obese patients for weight loss treatment.[9] Recent reports by the Academy of Medical Royal Colleges (AMRC) and the Royal College of Physicians (RCP) have called for similar action in the UK.[10,11] Under the policy of 'making every contact count,' the AMRC recommended that health professionals should routinely speak to overweight and obese patients about diet and exercise habits at each appointment and offer help. This recommendation is also made directly to health professionals in the UK via NICE guidelines.[12]

In spite of these recommendations, current figures indicate that only a minority of health professionals routinely give weight advice.[13–18] A range of barriers, including perceived lack of time, inadequate knowledge, lack of training or confidence and inadequate teaching materials have been identified as contributing to the relatively low rates of weight control advice from health professionals.[19–25] Importantly, many health professionals also doubt the usefulness of providing weight advice because they don't feel it will change patients' behaviour.[19,21,24,26]

However, there is evidence suggesting that health professionals can play a valuable role in helping patients to change their behaviour. Advice and involvement of health professionals in helping smokers quit is effective, and has led to evidence-based guidelines for their active intervention.[27] A recent systematic review and meta-analysis of US studies concluded that weight loss advice from a health professional in primary care is associated with positive weight loss behaviour change in overweight and obese patients,[28] but no equivalent studies have been conducted in the UK population.

This study therefore tested the hypothesis that advice to lose weight from a doctor or other health professional would be associated with a greater likelihood of trying to lose weight in a British sample of overweight and obese adults.

For peer review only - http://bmjopen.bmj.com/site/about/guidelines.xhtml

For peer review only

METHODS

Design and participants

Data on weight loss motivation and weight loss advice were collected from a large sample of British adults (n=1,986; 932 men, 1,054 women) as part of a home-based, face-to-face survey from across Great Britain in April 2012. To reduce potential bias, data were collected by an independent market research company (TNS) that had no knowledge of our study aims and who asked these questions alongside questions on other topics. TNS uses a random location methodology based on the 2001 Census small-area statistics and the postcode address file, stratified by Government Office Region and social grade, to select sample points. At each location, quotas are set for age, gender, children in the home, and working status to ensure a balanced sample of adults within effective contacted addresses. Interviews are carried out on weekdays between 2pm and 8pm and at the weekend. Interviewers are instructed to leave three doors between each successful interview.

This survey was designed as part of an ongoing study assessing changes in weight perceptions in the British population (see Johnson et al. [29] for findings of the previous two surveys in this series), but it seemed timely given recent recommendations to also use the data collected to explore the relationship between health professional advice to lose weight and weight loss motivation. The majority of respondents provided height and weight data (n=1,557). These analyses focus on respondents with a BMI in the overweight or obese range (BMI ≥25; n=810).

Measures

Weight loss motivation: Respondents were asked how much they would ideally like to weigh, with desire to weigh less indexed by reporting an ideal weight ≤95% of self-reported current weight (i.e. ≥5% weight loss). They were also asked which of the following statements best described them: 1) *I'm not bothered about my weight*; 2) *I watch my weight to keep it where it is now*; 3) *I'm trying to lose weight*; or 4) *I'm trying to gain weight*. Respondents indicating they were trying to lose weight were compared with the other groups combined.

Health professional advice to lose weight: Respondents were asked whether a doctor or other health professional had ever told them that they should lose weight (yes/no).

Current anthropometry: Height and weight were self-reported in metric or imperial units as the respondent preferred. BMI (kg/m^2) was calculated from self-reported heights and weights.

For peer review only - http://bmjopen.bmj.com/site/about/guidelines.xhtml

For peer review only

Demographics: Age, sex, ethnicity, marital status, and socioeconomic status (SES) were included in the analyses. Due to the small number of participants from any individual ethnic minority group, ethnicity was only categorised as white vs. non-white. Marital status was categorised as married/living as married vs. unmarried. SES was defined according to the National Readership Survey classification of the person's current or last occupation,[30] and dichotomised into ABC1 (higher SES) vs. C2DE (lower SES).

Statistical analyses

Analyses were done using IBM SPSS Statistics version 19, with weighted data to match the population characteristics. Variables used for weighting included age, sex, social grade, and standard region. Analyses were repeated on unweighted data with no significant differences in the results, so with the exception of the sample description, only analyses on weighted data are reported.

Descriptive statistics (unweighted data) are presented for sample characteristics and prevalence of weight loss motivation and weight loss recommendations. Prevalence statistics are also reported for normal weight (BMI 18.5-24.9) respondents in the sample (n=705) for comparison. Multivariable logistic regression (weighted data) was used to examine associations between health professional advice to lose weight and weight loss motivation controlling for age, sex, ethnicity, marital status, SES, and weight status (overweight vs. obese).

There was very little missing data (0.4% (n=3) for health professional advice, 9.4% (n=76) for desire to weigh less, 1.7% (n=14) for attempting to lose weight, 0% (n=0) for demographic variables). Analyses were run only for participants who had provided full data on the relevant variables (n=731, 90.2% for analyses of desire to weigh less, n=793, 97.9% for analyses on attempting to lose weight).

RESULTS

Weight and height data were provided by 1,557 out of 1,986 respondents, of whom 48 (3%) had a BMI within the underweight range (BMI <18.5), 699 (45%) were in the healthy weight range, 528 (34%) were overweight (BMI ≥25 and <30), and a further 282 (18%) were obese (BMI ≥30). Subsequent analyses are restricted to respondents whose BMI defined them as overweight or obese (n=810).

For peer review only - http://bmjopen.bmj.com/site/about/guidelines.xhtml

Demographic and anthropometric characteristics of the overweight and obese respondents are shown in Table 1. Their mean age was 51.3 years (range: 16 to 90 years), 47% were female, 89% were white, 62% were married or living as married, and 61% were lower SES. Their mean weight was 85.3kg and mean BMI was 29.6kg/m^2 (27.2kg/m^2 in overweight respondents and 34.2kg/m^2 in obese respondents).

A quarter (26%) of the overweight or obese respondents (17% of overweight and 42% of obese) reported ever having received health professional advice to lose weight. For comparison, the rate of health professional advice among normal weight respondents in the sample (n=705) was 4%. Among those who were overweight or obese, mean BMI was higher among those who had received health professional advice to lose weight (32.1kg/m^2, SD 5.2) than had not (28.7kg/m^2, SD 3.5; $t(276.93) = 8.60$, $P<0.001$). Health professional advice appeared to become normative (i.e. was reported by more than 50% of respondents) at a BMI of approximately 37kg/m^2 (see Figure 1).

Overall, 68% of respondents wanted to weigh less and 45% were currently trying to lose weight. These rates are notably higher than were reported by normal weight respondents, among whom 21% wanted to weigh less and 13% were trying to lose weight. Having received health professional advice to lose weight was associated with higher prevalence of wanting to weigh less (89% vs. 61%) and of attempting weight loss (68% vs. 37%) in the overweight/obese sample. This pattern was the same for both obese and overweight participants (see Figure 2).The results of the multivariable logistic regression models are shown in Table 2. Compared with overweight respondents, obese respondents were more likely to want to lose weight (OR=8.57) and also more likely to be attempting to lose weight (OR=1.91). Being female quadrupled the odds of wanting to weigh less (OR=4.39) and doubled the odds of weight loss attempts (OR=1.93). The odds of attempting weight loss were significantly lower in older respondents (OR=0.52) and higher in higher SES respondents (OR=1.40), but there was no significant association with marital status or ethnicity. After controlling for the effects of demographics and weight status, having received health professional advice to lose weight more than tripled the odds of both wanting to weigh less (OR=3.71) and trying to lose weight (OR=3.53).

DISCUSSION

In this population-based sample of overweight and obese British adults, only a quarter (26%) reported having received advice to lose weight from a health professional. Around two-thirds reported a desire to weigh at least 5% less than their current weight, and just under half said they

For peer review only - http://bmjopen.bmj.com/site/about/guidelines.xhtml

were actively trying to lose weight. Having received health professional advice to lose weight was strongly associated with both wanting to weigh less and trying to lose weight after controlling for demographic characteristics.

Healthcare providers in primary care in the UK are required to record BMI on all patients, and recommended to discuss diet and exercise with all overweight and obese patients.[12] However, in this sample, fewer than half (42%) of obese respondents and only 17% of overweight respondents reported having been recommended by a doctor or other health professional to lose weight; findings similar to US studies.[13–18] Nonetheless, this is considerably higher than the 5% (among overweight respondents) and 16% (among obese respondents) reported in a similar British survey in 2001.[8] Health professional advice to lose weight became normative (i.e. observed in over 50% of respondents) at a BMI of 37. This is consistent with previous research showing that health professionals often do not pay attention to weight until a patient is at a much higher BMI than what is actually defined as obese.[31–33] Health professionals report an array of barriers to providing weight loss advice, including perceived lack of time, knowledge, training, and confidence.[19–25] Perhaps most importantly, obesity treatment is often perceived by physicians to be a daunting or even futile task,[26] and many say they find it professionally unrewarding.[34–38] This could well affect their enthusiasm to broach the topic.

Despite the notorious difficulty in achieving and maintaining significant weight loss, one study found that almost half of overweight and obese individuals believed they could lose weight if they felt they needed to.[21] This may highlight an important role for health professionals to give clear advice to patients when weight loss is needed. Our results demonstrate that receiving health professional advice to lose weight was associated with desiring a lower body weight, and more importantly, with attempting weight loss. Odds ratios were similar to those observed in a meta-analysis of US studies.[28] Among respondents who desired to weigh less, health professional advice was associated with being significantly more likely to be trying to lose weight. The translation of behavioural intentions to changes in behaviour is known to be a major block in lifestyle interventions.[39] The finding observed here suggests that in the case of weight loss, advice from a health professional can help to bridge the intention-behaviour gap.

Together, these results provide strong support for the recommendation that physicians and other health professionals should discuss weight with overweight and obese patients.[9,10] Targeted education and training programmes on weight counselling for health professionals could help overcome some of the barriers that hold them back from ‘making every contact count’.[10]

For peer review only - http://bmjopen.bmj.com/site/about/guidelines.xhtml

The findings of this study are subject to several limitations. We do not have response rate information because of the method of sampling, and it is not possible to know whether those who declined to participate differed from those who agreed to take part. However, as the primary focus is the association between weight loss advice and weight loss attempts, the sampling method is not likely to influence the findings. However, in terms of prevalence of weight loss advice and weight loss attempts, the results only reflect the experience of individuals who agreed to take part; which may overestimate levels of weight concern. Use of self-reported weights and heights mean that BMI is likely to be underestimated.[40,41] Consistent with this, the prevalence of obesity was lower than figures based on measured anthropometric data from the most recent Health Survey for England [42] (18% in the present study vs. 26% in the HSE). This may limit the extent to which findings can be generalised to the entire overweight and obese population. and results should therefore be interpreted as potentially applying to a population with a higher BMI but by an unknown amount.

The finding that only a minority of overweight and obese people had been advised to lose weight was important, but it is possible that more had received advice than recalled it. Health professional-reported rates of weight counselling are markedly higher than the 40-50% reported by obese patients,[43] although there is some evidence to suggest health professionals may overestimate the level of intervention they provide to patients.[43] Guiding health professionals on how to provide weight control advice in a way that resonates more strongly with their patients could improve the effectiveness of the advice and reduce any reservations about its utility.

These data do not address the reasons for being given or not given weight loss advice. People who had not received advice may not have seen a health professional since they became overweight or obese, they may have seen a health professional who did not identify their weight status, or the health professional may have recognised their overweight/obesity but had reasons not to mention it. In addition, we had no data on how recently participants had seen a health professional, or, for those who did report receiving advice, on the type of health professional it had come from (GP, hospital physician, nurse, dietitian, pharmacist, etc.). In Britain, patients typically visit their GP upon registering, and subsequently only when seeking help for a specific health concern (unless they require regular check-ups for a long-term medical condition). Patients who have never registered with a primary care physician (rather have only been to specialists or A&E), or patients who have not seen one in a long time, may not have had the opportunity to have weight loss addressed. Advice may be more or less effective coming from a certain type of health professional, but it was not possible to explore this using the data we had available. Identifying the determinants of giving weight loss advice could further help tailor health professional training.

For peer review only - http://bmjopen.bmj.com/site/about/guidelines.xhtml

There was also no information on how recently participants had received advice from a health professional to lose weight. It is unclear whether "ever" having received advice is important, or whether the timing of advice within the last week/month/year/decade was important. This is an avenue for exploration in future research.

The use of a cross-sectional design meant we were not able to determine whether health professional advice increased motivation to lose weight on an individual level. Individuals who are already concerned about their health may visit their doctors more often, and therefore have more opportunities to elicit weight loss advice, and they may even have asked for advice directly. The design also precluded assessment of weight loss success following health professional advice. Before the present results can be taken forward to guide policy, there needs to be evidence that advice from a health professional also leads to successful weight loss outcomes; and exploration of how the nature, extent, and cost of that advice, and from whom, relates to the extent and frequency of that success. Advice-only interventions have been shown to be less effective than more intensive interventions in producing positive weight loss outcomes [44], but previous research has demonstrated better long-term weight outcomes in treatment programmes following a medical trigger for weight loss,[28] and extrapolation from studies of GP advice for smoking cessation [27] give some cause for optimism. Prospective longitudinal research is needed to provide insight into motivational changes and actual weight reduction following advice to lose weight.

The results of this study confirm that many overweight and obese adults in Great Britain express a desire to weigh less than they do, but notably fewer are actively trying to lose weight. Advice from a health professional was strongly associated with attempting weight loss, supporting the recommendation that health professionals should discuss weight with overweight and obese patients as a matter of routine. Better training for health professionals in discussing weight issues could make a significant contribution to population weight management.

For peer review only - http://bmjopen.bmj.com/site/about/guidelines.xhtml

TABLES

Table 1 Demographic and anthropometric characteristics of a sample of overweight and obese British adults (n=810). Values are numbers (percentages) unless stated otherwise

**Demographic and healthcare characteristics**	
Age (years)	
Mean (SD)	51.3 (17.9)
<55	447 (55.2)
≥55	363 (44.8)
Sex	
Male	432 (53.3)
Female	378 (46.7)
Ethnicity	
White	721 (89.0)
Non-white	88 (10.9)
Marital status	
Unmarried	312 (38.5)
Married/living as married	498 (61.5)
SES	
Lower	493 (60.9)
Higher	317 (39.1)
Health professional advice to lose weight	
No	599 (74.0)
Yes	208 (25.7)
**Anthropometric characteristics**	
Mean (SD) height, cm	169.4 (10.7)
Mean (SD) weight, kg	85.3 (16.2)
Mean (SD) BMI, kg/m^2	29.6 (4.3)
Weight status	
Overweight	528 (65.2)
Obese	282 (34.8)

Unweighted data shown.
Numbers may not sum to total sample number, as some items were not answered by all participants.
Percentages were derived from the full sample and may therefore not sum to 100%.
SES, socioeconomic status.

For peer review only

For peer review only - http://bmjopen.bmj.com/site/about/guidelines.xhtml

Table 2 Multivariable logistic regression models predicting desire to weigh less and attempting to lose weight in a sample of overweight and obese British adults (n=810)

	Desire to weigh less			Attempting to lose weight		
**Characteristics**	**OR**	**95% CI**	***P***	**OR**	**95% CI**	***P***
Age (years)						
<55	1.00	-	-	1.00	-	-
≥55	0.84	0.57-1.23	0.369	0.52	0.38-0.71	<0.001
Sex						
Male	1.00	-	-	1.00	-	-
Female	4.39	2.94-6.56	<0.001	1.93	1.43-2.63	<0.001
Ethnicity						
White	1.00	-	-	1.00	-	-
Non-white	0.96	0.51-1.84	0.912	1.11	0.67-1.85	0.686
Marital status						
Unmarried	1.00	-	-	1.00	-	-
Married/living as married	0.87	0.59-1.29	0.483	1.34	0.98-1.84	0.065
SES						
Lower	1.00	-	-	1.00	-	-
Higher	1.34	0.92-1.95	0.125	1.40	1.03-1.89	0.030
Weight status						
Overweight	1.00	-	-	1.00	-	-
Obese	8.57	4.87-15.08	<0.001	1.91	1.38-2.64	<0.001
Health professional advice to lose weight						
No	1.00	-	-	1.00	-	-
Yes	3.71	2.10-6.55	<0.001	3.53	2.44-5.10	<0.001

Data were weighted on age, sex, social grade, and standard region. Results were not notably different when analyses were run on unweighted data.
All variables entered into the models are shown in the table; there were no additional covariates.
SES, socioeconomic status; OR, odds ratio; CI, confidence interval.

For peer review only

FIGURES

Figure 1 Percentage of overweight and obese British adults reporting ever having received health professional advice to lose weight by BMI

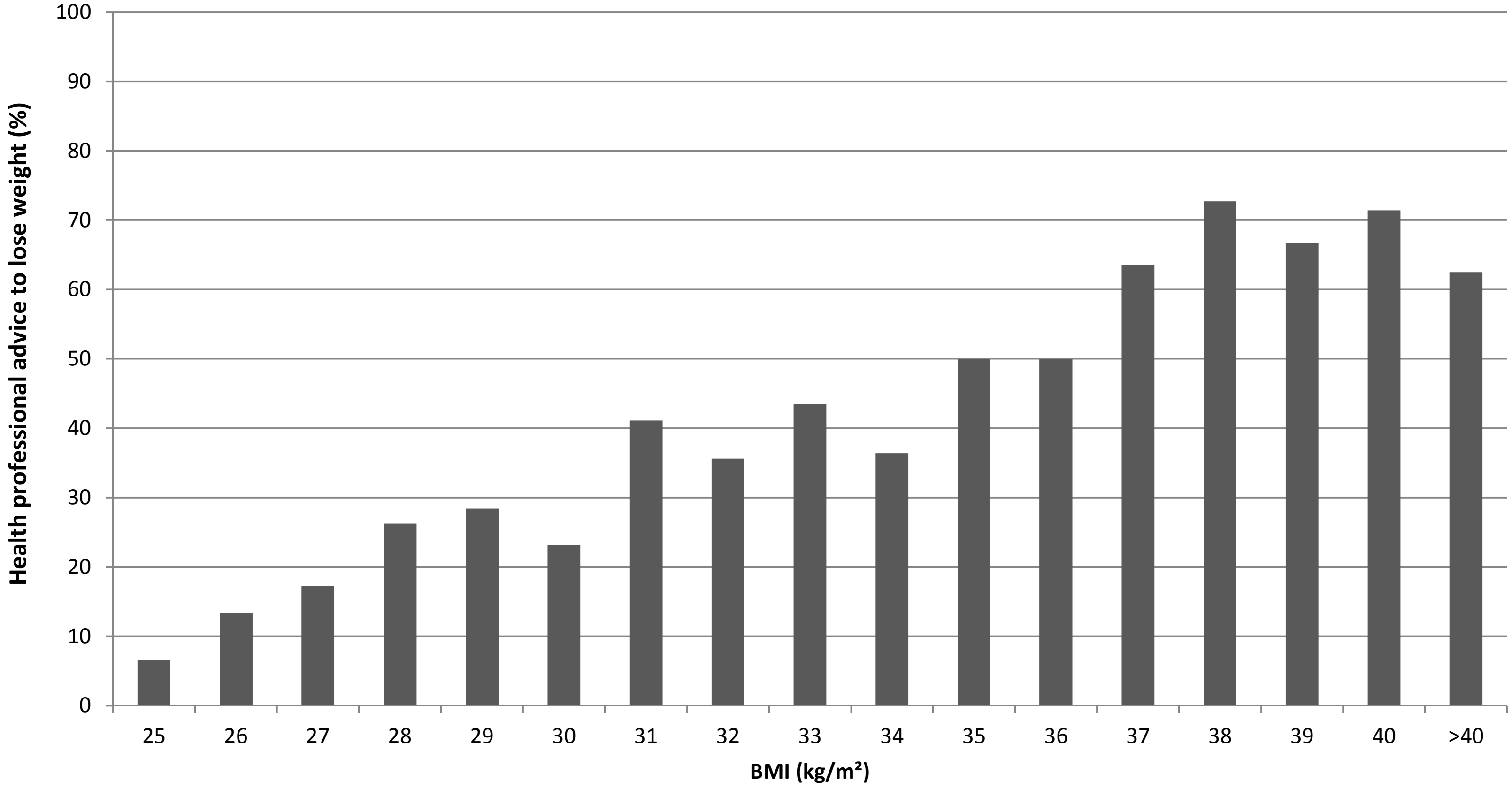

Unweighted data shown.
BMI points were rounded down such that a BMI point of 25 includes 25.0-25.9.

For peer review only - http://bmjopen.bmj.com/site/about/guidelines.xhtml

Figure 2 Prevalence of attempting to lose weight in overweight and obese British adults by health professional advice to lose weight (yes/no) and weight status (overweight/obese)

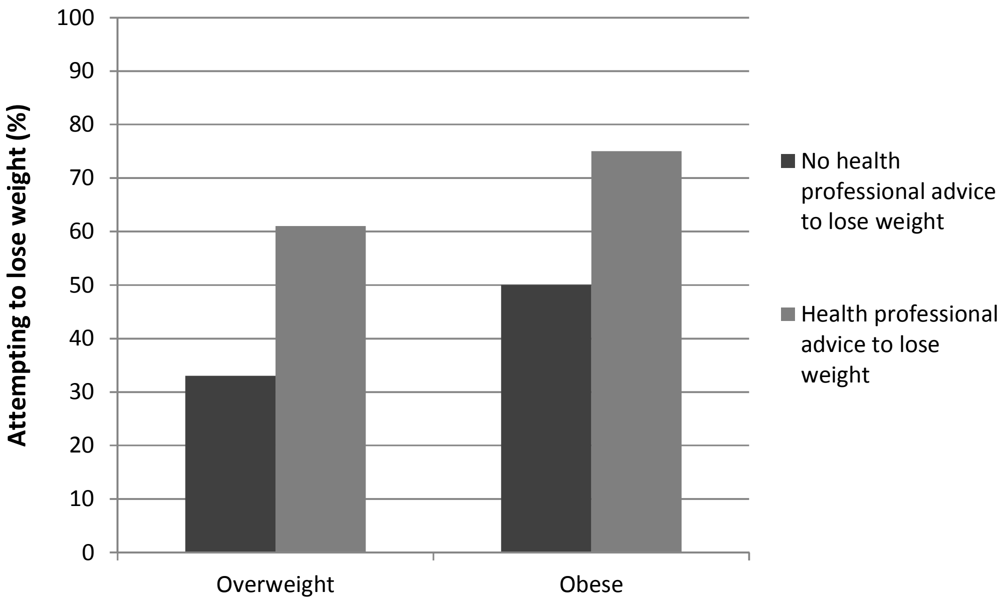

Unweighted data shown.

For peer review only - http://bmjopen.bmj.com/site/about/guidelines.xhtml

CONTRIBUTORSHIP STATEMENT

Everyone listed as an author fulfils all three of the ICMJE guidelines for authorship: 1) substantial contributions to conception and design, acquisition of data, or analysis and interpretation of data; 2) drafting the article or revising it critically for important intellectual content; and 3) final approval of the version to be published.

SJ, JW, FJ, NF, and RB were responsible for the study concept and design. JW obtained funding. JW and FJ acquired the data. SJ, JW, FJ, NF, and RB analysed and interpreted the data. SJ did the statistical analysis. SJ drafted the manuscript, and all authors revised it for important intellectual content. All authors had final approval of the version to be published. JW is the guarantor.

All authors had full access to all of the data (including statistical reports and tables) in the study and can take responsibility for the integrity of the data and the accuracy of the data analysis.

COMPETING INTERESTS

All authors have completed the Unified Competing Interest form at www.icmje.org/coi_disclosure.pdf (available on request from the corresponding author) and declare: support for the study by grants from the UK Medical Research Council, UK Economic and Social Research Council, and Cancer Research UK; no financial relationships with any organisations that might have an interest in the submitted work in the previous three years; and no other relationships or activities that could appear to have influenced the submitted work.

FUNDING

This study was supported by grants from the UK Medical Research Council, UK Economic and Social Research Council, and Cancer Research UK.

The study sponsors had no role in the study design; in the collection, analysis, and interpretation of the data; in the preparation, review, or approval of the manuscript; or in the decision to submit it for publication. The researchers were independent of the funding agencies.

For peer review only

REFERENCES

1. Guh DP, Zhang W, Bansback N, Amarsi Z, Laird Birmingham C, Anis AH. The incidence of co-morbidities related to obesity and overweight: A systematic review and meta-analysis. BMC Public Health 2009;**9**:88 (2009).
2. Ford ES, Mokdad AH. Epidemiology of obesity in the western hemisphere. JCEM 2008;**93**:S1–S8.
3. Blackburn, G. Effect of degree of weight loss on health benefits. Obes Res 1995;**3**:S211–S216.
4. Lavie CJ, Milani RV, Artham SM, Patel D, Ventura HO. The obesity paradox, weight loss, and coronary disease. Am J Med 2009;**122**:1106–1114.
5. Franz MJ, VanWormer JJ, Crain AL, Boucher JL, Histon T, Caplan W et al. Weight-loss outcomes: a systematic review and meta-analysis of weight-loss clinical trials with a minimum 1-year follow-up. J Am Diet Ass 2007;**107**:1755–1767.
6. Tsai AG, Wadden TA. Systematic review: an evaluation of major commercial weight loss programs in the United States. Ann Intern Med 2005;**142**:56–66.
7. Yaemsiri S, Slining MM, Agarwal SK. Perceived weight status, overweight diagnosis, and weight control among US adults: the NHANES 2003–2008 Study. Int J Obes 2011;**35**:1063–1070.
8. Wardle J, Johnson F. Weight and dieting: examining levels of weight concern in British adults. Int J Obes 2002;**26**:1144–1149.
9. Moyer VA. Screening for and management of obesity in adults: U.S. Preventive Services Task Force recommendation statement. Ann Intern Med 2012;**157**:373–378 (2012).
10. Academy of Medical Royal Colleges. Measuring Up: The medical profession's prescription for the nation's obesity crisis. Academy of Medical Royal Colleges, 2013. http://www.aomrc.org.uk/projects/obesity-steering-group.html (accessed 17 Jun 2013).
11. Royal College of Physicians. The training of health professionals for the prevention and treatment of overweight and obesity. Royal College of Physicians, 2010. http://www.rcplondon.ac.uk/sites/default/files/press_releases/2010/12/obesity-report-2010.pdf (accessed 17 Jun 2013).
12. National Institute for Health and Clinical Excellence (NICE). Obesity: the prevention, identification, assessment and management of overweight and obesity in adults and children. NICE, 2006. http://www.nice.org.uk/ (accessed 17 Jun 2013).
13. Clune A, Fischer JG, Lee JS, Reddy S, Johnson MA, Hausman DB. Prevalence and predictors of recommendations to lose weight in overweight and obese older adults in Georgia senior centers. Prev Med 2010;**51**:27–30.
14. Galuska DA, Will JC, Serdula MK, Ford ES. Are health care professionals advising obese patients to lose weight? JAMA 1999;**282**:1576–1578.
15. Jackson JE, Doescher MP, Saver BG, Hart LG. Trends in professional advice to lose weight among obese adults, 1994 to 2000. J Gen Intern Med 2005;**20**:814–818.
16. Loureiro ML, Nayga Jr RM. Obesity, weight loss, and physician's advice. Soc Sci Med 2006;**62**:2458–2468.
17. Sciamanna C, Tate D, Lang W, Wing RR. Who reports receiving advice to lose weight?: Results from a multistate survey. Arch Intern Med 2000;**160**:2334–2339.

For peer review only - http://bmjopen.bmj.com/site/about/guidelines.xhtml

18. Stafford RS, Farhat JH, Misra B, Schoenfeld DA. National patterns of physician activities related to obesity management. Arch Fam Med 2000;**9**:631–638.
19. Hiddink GJ, Hautvast JG, Van Woerkum CM, Fieren CJ, Van 't Hof MA. Nutrition guidance by primary-care physicians: perceived barriers and low involvement. Eur J Clin Nutr 1995;**49**:842–851.
20. Harris JE, Hamaday V, Mochan E. Osteopathic family physicians' attitudes, knowledge, and self-reported practices regarding obesity. J Am Osteopath Assoc 1999;**99**:358–365.
21. Ruelaz AR, Diefenbach P, Simon B, Lanto A, Arterburn D, Shekelle PG. Perceived barriers to weight management in primary care--perspectives of patients and providers. J Gen Intern Med 2007;**22**:518–522.
22. Kenner MM, Taylor ML, Dunn PC, Gruchow HW, Kolasa K. primary care providers need a variety of nutrition and wellness patient education materials. J Am Diet Assoc 1999;**99**:462–466.
23. Leverence RR, Williams RL, Sussman A, Crabtree BF. Obesity counseling and guidelines in primary care: a qualitative study. Am J Prev Med 2007;**32**:334–339.
24. Foster GD, Wadden TA, Makris AP, Davidson D, Sanderson RS, Allison DB et al. Primary care physicians' attitudes about obesity and its treatment. Obes Res 2003;**11**:1168–1177.
25. Michie S. Talking to primary care patients about weight: A study of GPs and practice nurses in the UK. Psychol Health Med 2007;**12**:521–525.
26. Frank A. Futility and avoidance. Medical professionals in the treatment of obesity. JAMA 1993;**269**:2132–2133.
27. West R, McNeill A, Raw M. Smoking cessation guidelines for health professionals: an update. Thorax 2000;**55**:987–999.
28. Rose SA, Poynter PS, Anderson JW, Noar SM, Conigliaro J. Physician weight loss advice and patient weight loss behavior change: a literature review and meta-analysis of survey data. Int J Obes 2013;**37**:118-128.
29. Johnson F, Cooke L, Croker H, Wardle J. Changing perceptions of weight in Great Britain: comparison of two population surveys. BMJ 2008;**337**:a494–a494.
30. National Readership Survey. Social grade - definitions and discriminatory power. National Readership Survey, 2007. http://www.nrs.co.uk/lifestyle.html (accessed 17 Jun 2013).
31. Bardia A, Holtan SG, Slezak JM, Thompson WG. Diagnosis of obesity by primary care physicians and impact on obesity management. Mayo Clin Proc 2007;**82**:927–932.
32. Huang J, Yu H, Marin E, Brock S, Carden D, Davis T. Physicians' weight loss counseling in two public hospital primary care clinics. Acad Med 2004;**79**:156–161.
33. O'Brien SH, Holubkov R, Reis EC. Identification, evaluation, and management of obesity in an academic primary care center. Pediatrics 2004;**114**:e154–e159.
34. Bocquier A, Verger P, Basdevant A, Andreotti G, Baretge J, Villani P et al. Overweight and obesity: knowledge, attitudes, and practices of general practitioners in France. Obes Res 2005;**13**:787–795.
35. Campbell K, Crawford D. Management of obesity: attitudes and practices of Australian dietitians. Int J Obes Relat Metab Disord 2000;**24**:701–710.
36. Campbell K, Engel H, Timperio A, Cooper C, Crawford D. Obesity management: Australian general practitioners' attitudes and practices. Obes Res 2000;**8**:459–466.
37. Loomis GA, Connolly KP, Clinch CR, Djuric DA. Attitudes and practices of military family physicians regarding obesity. Mil Med 2001;**166**:121–125.

For peer review only

38. Thuan JF, Avignon A. Obesity management: attitudes and practices of French general practitioners in a region of France. Int J Obes 2005;**29**:1100–1106.

39. Webb TL, Sheeran P. Does changing behavioral intentions engender behavior change? A meta-analysis of the experimental evidence. Psychol Bull 2006;**132**:249–268.

40. Cameron R, Evers SE. Self-report issues in obesity and weight management: State of the art and future directions. Behav Assess 1990. http://psycnet.apa.org/psycinfo/1990-18712-001 (accessed 17 Jun 2013).

41. Roberts RJ. Can self-reported data accurately describe the prevalence of overweight? Public Health 1995;**109**:275–284.

42. Health Survey for England. Health Survey for England - 2010: Trend tables. Health Survey for England, 2010. http://www.ic.nhs.uk/statistics-and-data-collections/health-and-lifestyles-related-surveys/health-survey-for-england/health-survey-for-england--2010-trend-tables (accessed 17 Jun 2013).

43. Laws R. Current approaches to obesity management in UK Primary Care: the Counterweight Programme. J Hum Nutr Diet 2004;**17**:183–190.

44. Franz MJ, VanWormer JJ, Crain AL, Boucher JL, Histon T, Caplan W et al. Weight-loss outcomes: a systematic review and meta-analysis of weight-loss clinical trials with a minimum 1-year follow-up. J Am Diet Ass 2007;**107**:1755–1767.

For peer review only

For peer review only - http://bmjopen.bmj.com/site/about/guidelines.xhtml

The impact of a health professional recommendation on weight loss attempts in overweight and obese British adults: a cross-sectional analysis

Sarah E Jackson, Jane Wardle, Fiona Johnson, Nicholas Finer, Rebecca J Beeken

Department of Epidemiology and Public Health, University College London, London WC1E 6BT, UK

Sarah E Jackson

PhD student

Jane Wardle

Professor of Clinical Psychology

Fiona Johnson

Research Psychologist

Rebecca J Beeken

Senior Research Psychologist

UCL Institute of Cardiovascular Science, 170 Tottenham Court Road, London W1T 7HA, UK

Nicholas Finer

Honorary Professor

Correspondence to: RJ Beeken. Email: r.beeken@ucl.ac.uk. Telephone: +44 (0)20 7679 1720.

Keywords weight loss; lose weight; health professional advice; doctor advice; primary care.

Word count 2,~~377~~856

For peer review only

ABSTRACT

Objectives To examine the effect that health professional (HP) advice to lose weight has on overweight and obese adults' motivation to lose weight and attempts to lose weight.

Design Cross-sectional survey.

Setting Great Britain.

Participants 810 overweight or obese (BMI ≥25kg/m^2) adults.

Main outcome measures Participants were asked if they had ever received HP advice to lose weight and reported their desire to weigh less (~~preferred~~ ideal weight ≤95% of current weight) and whether they were attempting to lose weight.

Results Only 17% of overweight and 42% of obese respondents recalled ever having received HP advice to lose weight. HP advice was associated with wanting to weigh less (89% vs. 61% among those not receiving advice) and attempting to lose weight (68% vs. 37%). In multivariable analyses, HP advice to lose weight was associated with increased odds of wanting to weigh less (odds ratio (OR) = 3.71, 95% CI = 2.10–6.55) and attempting to lose weight (OR = 3.53, 95% CI = 2.44–5.10) independent of demographic characteristics and weight status.

Conclusions HP advice to lose weight appears to increase motivation to lose weight and weight loss behaviour, but only a minority of overweight or obese adults receive such advice. Better training for HPs in delivering brief weight counselling could offer an opportunity to improve obese patients' motivation to lose weight.

For peer review only

ARTICLE SUMMARY

Article focus

- Health professionals are recommended to routinely speak to overweight and obese patients about their weight and offer weight control advice, but many doubt the effectiveness of providing advice.
- This study examined associations between health professional advice for weight loss and patients' motivation to lose weight in a British sample of overweight and obese adults.

Key messages

- Overweight and obese individuals who have received health professional advice to lose weight are over three times as likely to attempt weight loss as those who have never received advice.

Strengths and limitations

- This is the first study to show an association between health professional advice to lose weight and weight loss attempts in a British sample.
- Data were not collected on actual weight loss so the impact of health professional advice on weight loss outcomes could not be established.

For peer review only

For peer review only - http://bmjopen.bmj.com/site/about/guidelines.xhtml

INTRODUCTION

In most Western countries, between 40% and 70% of adults have a body weight that places them at risk of developing weight-related long-term health problems.[1,2] There is good evidence that losing as little as 5% of body weight confers significant cardio-metabolic benefit for overweight and obese individuals,[3,4] although sustained weight loss is difficult to achieve.[5,6]

The majority of overweight and obese adults say they want to lose weight,[7] but only around half report actively trying to lose weight.[7,8] One potential source of motivation to lose weight is advice from a health professional. Physicians and other health professionals have a unique opportunity to raise obese patients' awareness of their weight status and its associated health risks, and to offer advice on weight management. In the US, doctors are recommended to screen for obesity and refer all obese patients for weight loss treatment.[9] Recent reports by the Academy of Medical Royal Colleges (AMRC) and the Royal College of Physicians (RCP) have called for similar action in the UK.[10,11] Under the policy of 'making every contact count,'~~,~~ the AMRC recommended that health professionals should routinely speak to overweight and obese patients about diet and exercise habits at each appointment and offer help. This recommendation is also made directly to health professionals in the UK via NICE ~~and SIGN~~ guidelines~~, and through the quality and outcomes framework (QOF)~~.[12~~–14~~]

In spite of these recommendations, current figures indicate that only a minority of health professionals routinely give weight advice.[~~15~~13–~~20~~18] A range of barriers, including perceived lack of time, inadequate knowledge, lack of training or confidence and inadequate teaching materials have been identified as contributing to the relatively low rates of weight control advice from health professionals.[~~21~~19–~~27~~25] Importantly, many health professionals also doubt the usefulness of providing weight advice because they don't feel it will change patients' behaviour.[~~21~~19,~~23~~21,~~26~~24,~~28~~26]

However, there is evidence suggesting that health professionals can play a valuable role in helping patients to change their behaviour. Advice and involvement of health professionals in helping smokers quit is effective, and has led to evidence-based guidelines for their active intervention.[~~29~~27] A recent systematic review and meta-analysis of US studies concluded that weight loss advice from a health professional in primary care is associated with positive weight loss behaviour change in overweight and obese patients,[~~30~~28] but no equivalent studies have been conducted in the UK population.

For peer review only - http://bmjopen.bmj.com/site/about/guidelines.xhtml

This study therefore tested the hypothesis that advice to lose weight from a doctor or other health professional would be associated with a greater likelihood of trying to lose weight in a ~~UK~~ British sample of overweight and obese adults.

METHODS

Design and participants

Data on weight loss motivation and weight loss advice were collected from a large sample of British adults (n=1,986~~,~~; 932 men, 1,054 women) as part of a home-based, face-to-face survey from across Great Britain in April 2012. To reduce potential bias, data were collected by an independent market research company (TNS) that had no knowledge of our study aims and who asked these questions alongside questions on other topics. TNS uses a random location methodology based on the 2001 Census small-area statistics and the postcode address file, stratified by Government Office Region and social grade, to select sample points. At each location, quotas are set for age, gender, children in the home, and working status to ensure a balanced sample of adults within effective contacted addresses. Interviews are carried out on weekdays between 2pm and 8pm and at the weekend. Interviewers ~~were~~ are instructed to leave three doors between each successful interview.

This survey was designed as part of an ongoing study assessing changes in weight perceptions in the British population (see Johnson et al. [~~31~~29] for findings of the previous two surveys in this series), but it seemed timely given recent recommendations to also use the data collected to explore the relationship between health professional advice to lose weight and weight loss motivation. The majority of respondents provided height and weight data (n=1,557). These analyses focus on respondents with a BMI in the overweight or obese range (BMI ≥25; n=810).

For peer review only

Measures

Weight loss motivation: Respondents were asked how much they would ideally like to weigh, with desire to weigh less indexed by reporting an ideal weight ≤95% of self-reported current weight (i.e. ≥5% weight loss). They were also asked which of the following statements best described them: 1) *I'm not bothered about my weight*; 2) *I watch my weight to keep it where it is now*; 3) *I'm trying to lose weight*; or 4) *I'm trying to gain weight*. Respondents indicating they were trying to lose weight were compared with the other groups combined.

For peer review only - http://bmjopen.bmj.com/site/about/guidelines.xhtml

Health professional advice to lose weight: Respondents were asked whether a doctor or other health professional had ever told them that they should lose weight (yes/no).

Current anthropometry: Height and weight were self-reported in metric or imperial units as the respondent preferred. BMI (kg/m^2) was calculated from self-reported heights and weights.

Demographics: Age, sex, ethnicity, marital status, and socioeconomic status (SES) were included in the analyses. Due to the small number of participants from any individual ethnic minority group, ethnicity was only categorised as white vs. non-white. Marital status was categorised as married/living as married vs. unmarried. SES was defined according to the National Readership Survey classification of the person's current or last occupation,[~~32~~30] and dichotomised into ABC1 (higher SES) vs. C2DE (lower SES).

Statistical analyses

Analyses were done using IBM SPSS Statistics version 19, with weighted data to match the population characteristics. Variables used for weighting included age, sex, social grade, and standard region. Analyses were repeated on unweighted data with no significant differences in the results, so with the exception of the sample description, only analyses on weighted data are reported.

Descriptive statistics (unweighted data) are presented for sample characteristics and prevalence of weight loss motivation and weight loss recommendations. Prevalence statistics are also reported for normal weight (BMI 18.5-24.9) respondents in the sample (n=705) for comparison. Multivariable logistic regression (weighted data) was used to examine associations between health professional advice to lose weight and weight loss motivation controlling for age, sex, ethnicity, marital status, SES, and weight status (overweight vs. obese).

There was very little missing data (0.4% (n=3) for health professional advice, 9.4% (n=76) for desire to weigh less, 1.7% (n=14) for attempting to lose weight, 0% (n=0) for demographic variables). Analyses were run only for participants who had provided full data on the relevant variables (n=731, 90.2% for analyses of desire to weigh less, n=793, 97.9% for analyses on attempting to lose weight).

RESULTS

Weight and height data were provided by 1,557 out of 1,986 respondents, of whom 48 (3%) had a BMI within the underweight range (BMI <18.5), 699 (45%) were in the healthy weight range, 528

For peer review only - http://bmjopen.bmj.com/site/about/guidelines.xhtml

(34%) were overweight (BMI ≥25 and <30), and a further 282 (18%) were obese (BMI ≥30). Subsequent analyses are restricted to respondents whose BMI defined them as overweight or obese (n=810).

Demographic and anthropometric characteristics of the overweight and obese respondents are shown in Table 1. Their mean age was 51.3 years (range: 16 to 90 years), 47% were female, 89% were white, 62% were married or living as married, and 61% were lower SES. Their mean weight was 85.3kg and mean BMI was 29.6kg/m^2 (27.2kg/m^2 in overweight respondents and 34.2kg/m^2 in obese respondents).

~~Overall, a~~A quarter (26%) of the overweight or obese respondents (17% of overweight and 42% of obese) reported ever having received health professional advice to lose weight. For comparison, the rate of health professional advice among normal weight respondents in the sample (n=705) was 4%. Among those who were overweight or obese, ~~M~~mean BMI was higher among those who had received health professional advice to lose weight (32.1kg/m^2, SD 5.2) than had not (28.7kg/m^2, SD 3.5; $t(276.93) = 8.60$, $P<0.001$). Health professional advice appeared to become normative (i.e. was reported by more than 50% of respondents) at a BMI of approximately 37kg/m^2 (see Figure 1).

Overall, 68% of respondents wanted to weigh less~~,~~ and 45% were currently trying to lose weight. These rates are notably higher than were reported by normal weight respondents, among whom 21% wanted to weigh less and 13% were trying to lose weight. ~~Receiving~~ Having received health professional advice to lose weight was associated with higher prevalence of wanting to weigh less (89% vs. 61%) and of attempting weight loss (68% vs. 37%) in the overweight/obese sample. This pattern was the same for both obese and overweight participants (see Figure 2).

The results of the multivariable logistic regression models are shown in Table 2. Compared with overweight respondents, obese respondents were more likely to want to lose weight (OR=8.57) and also more likely to be attempting to lose weight (OR=1.91). Being female quadrupled the odds of wanting to weigh less (OR=4.39) and doubled the odds of weight loss attempts (OR=1.93). The odds of attempting weight loss were significantly lower in older respondents (OR=0.52) and higher in higher SES respondents (OR=1.40), but there was no significant association with marital status or ethnicity. After controlling for the effects of demographics and weight status, having received health professional advice to lose weight more than tripled the odds of both wanting to weigh less (OR=3.71) and trying to lose weight (OR=3.53).

For peer review only - http://bmjopen.bmj.com/site/about/guidelines.xhtml

DISCUSSION

In this population-based sample of overweight and obese British adults, only a quarter (26%) reported having received advice to lose weight from a health professional. Around two-thirds reported a desire to weigh at least 5% less than their current weight, and just under half said they were actively trying to lose weight. Having received health professional advice to lose weight was strongly associated with both wanting to weigh less and trying to lose weight after controlling for demographic characteristics.

Healthcare providers in primary care in the UK are required to record BMI on all patients, and recommended to discuss diet and exercise with all overweight and obese patients.[12~~–14~~] However, in this sample, fewer than half (42%) of obese respondents and only 17% of overweight respondents reported having been recommended by a doctor or other health professional to lose weight; findings similar to US studies.[~~15~~13–~~20~~18] Nonetheless, this is considerably higher than the 5% (among overweight respondents) and 16% (among obese respondents) reported in a similar British survey in 2001.[8] Health professional advice to lose weight became normative (i.e. observed in over 50% of respondents) at a BMI of 37. This is consistent with previous research showing that health professionals often do not pay attention to weight until a patient is at a much higher BMI than what is actually defined as obese.[31–33] Health professionals report an array of barriers to providing weight loss advice, including perceived lack of time, knowledge, training, and confidence.[~~21~~19–~~27~~25] Perhaps most importantly, obesity treatment is often perceived by physicians to be a daunting or even futile task,[~~28~~26] and many say they find it professionally unrewarding.[~~33~~34–~~37~~38] This could well affect their enthusiasm to broach the topic.

Despite the notorious difficulty in achieving and maintaining significant weight loss, one study found that almost half of overweight and obese individuals believed they could lose weight if they felt they needed to.[~~23~~21] This may highlight an important role for health professionals to give clear advice to patients when weight loss is needed. Our results demonstrate that receiving health professional advice to lose weight was associated with desiring a lower body weight, and more importantly, with attempting weight loss. Odds ratios were similar to those observed in a meta-analysis of US studies.[~~30~~28] Among respondents who desired to weigh less, health professional advice was associated with being significantly more likely to be trying to lose weight. The translation of behavioural intentions to changes in behaviour is known to be a major block in lifestyle interventions.[~~38~~39] The finding observed here suggests that in the case of weight loss, advice from a health professional can help to bridge the intention-behaviour gap.

For peer review only

For peer review only - http://bmjopen.bmj.com/site/about/guidelines.xhtml

Together, these results provide strong support for the recommendation that physicians and other health professionals should discuss weight with overweight and obese patients.[9,10] Targeted education and training programmes on weight counselling for health professionals could help overcome some of the barriers that hold them back from 'making every contact count'.[10]

The findings of this study are subject to several limitations. We do not have response rate information because of the method of sampling, and it is not possible to know whether those who declined to participate differed from those who agreed to take part. However, ~~but~~ as the primary focus is the association between weight loss advice and weight loss attempts, the sampling method is not likely to influence the findings. However, in terms of prevalence of weight loss advice and weight loss attempts, the results only reflect the experience of individuals who agreed to take part; which may overestimate levels of weight concern. Use of self-reported weights and heights mean that BMI is likely to be underestimated.[~~39~~40,~~40~~41] Consistent with this, the prevalence of obesity was lower than figures based on measured anthropometric data from the most recent Health Survey for England [~~41~~42] (18% in the present study vs. 26% in the HSE). This may limit the extent to which findings can be generalised to the entire overweight and obese population.~~,~~ and results should therefore be interpreted as potentially applying to a population with a higher BMI but by an unknown amount.

The finding that only a minority of overweight and obese people had been advised to lose weight was important, but it is possible that more had received advice than recalled it. Health professional-reported rates of weight counselling are markedly higher than the 40-50% reported by obese patients,[~~42~~43] although there is some evidence to suggest health professionals may overestimate the level of intervention they provide to patients.[~~42~~43] Guiding health professionals on how to provide weight control advice in a way that resonates more strongly with their patients could improve the effectiveness of the advice and reduce any reservations about its utility.

These data do not address the reasons for being given or not given weight loss advice. People who had not received advice may not have seen a health professional since they became overweight or obese, they may have seen a health professional who did not identify their weight status, or the health professional may have recognised their overweight/obesity but had reasons not to mention it. In addition, we had no data on how recently participants had seen a health professional, or, for those who did report receiving advice, on the type of health professional it had come from (GP, hospital physician, nurse, dietitian, pharmacist, etc.). In Britain, patients typically visit their GP upon registering, and subsequently only when seeking help for a specific health concern (unless they require regular check-ups for a long-term medical condition). Patients who have never registered

For peer review only - http://bmjopen.bmj.com/site/about/guidelines.xhtml

with a primary care physician (rather have only been to specialists or A&E), or patients who have not seen one in a long time, may not have had the opportunity to have weight loss addressed. Advice may be more or less effective coming from a certain type of health professional, but it was not possible to explore this using the data we had available. Identifying the determinants of giving weight loss advice could further help tailor health professional training.

There was also no information on how recently participants had received advice from a health professional to lose weight. It is unclear whether "ever" having received advice is important, or whether the timing of advice within the last week/month/year/decade was important. This is an avenue for exploration in future research.

The use of a cross-sectional design meant we were not able to determine whether health professional advice increased motivation to lose weight on an individual level. Individuals who are already concerned about their health may visit their doctors more often, and therefore have more opportunities to elicit weight loss advice, and they may even have asked for advice directly. The design also precluded assessment of weight loss success following health professional advice,. Before the present results can be taken forward to guide policy, there needs to be evidence that advice from a health professional also leads to successful weight loss outcomes; and exploration of how the nature, extent, and cost of that advice, and from whom, relates to the extent and frequency of that success. Advice-only interventions have been shown to be less effective than more intensive interventions in producing positive weight loss outcomes [44], but previous research has demonstrated better long-term weight outcomes in treatment programmes following a medical trigger for weight loss,[3028] and extrapolation from studies of GP advice for smoking cessation [2927] give some cause for optimism. Prospective longitudinal research is needed to provide insight into motivational changes and actual weight reduction following advice to lose weight.

The results of this study confirm that many overweight and obese adults in the UKGreat Britain express a desire to weigh less than they do, but notably fewer are actively trying to lose weight. Advice from a health professional was strongly associated with attempting weight loss, supporting the recommendation that health professionals should discuss weight with overweight and obese patients as a matter of routine. Better training for health professionals in discussing weight issues could make a significant contribution to population weight management.

For peer review only

For peer review only - http://bmjopen.bmj.com/site/about/guidelines.xhtml

TABLES

Table 1 ~~Sample d~~Demographic and anthropometric characteristics ~~among~~ of a sample of overweight and obese ~~respondents~~ British adults (n=810). Values are numbers (percentages) unless stated otherwise

**Demographic and healthcare characteristics**	
Age (years)	
Mean (SD)	51.3 (17.9)
<55	447 (55.2)
≥55	363 (44.8)
Sex	
Male	432 (53.~~5~~3)
Female	378 (46.7)
Ethnicity	
White	721 (89.0)
Non-white	88 (10.9)
Marital status	
Unmarried	312 (38.5)
Married/living as married	498 (61.5)
SES	
Lower	493 (60.9)
Higher	317 (39.1)
Health professional advice to lose weight	
No	599 (74.0)
Yes	208 (25.7)
**Anthropometric characteristics**	
Mean (SD) height, cm	169.4 (10.7)
Mean (SD) weight, kg	85.3 (16.2)
Mean (SD) BMI, kg/m^2	29.6 (4.3)
Weight status	
Overweight	528 (65.2)
Obese	282 (34.8)

~~SES = socio-economic status. Note:~~ Unweighted data shown.
Numbers may not sum to total sample number, as some items were not answered by all participants.
Percentages were derived from the full sample and may therefore not sum to 100%.
SES, socioeconomic status.

For peer review only

For peer review only - http://bmjopen.bmj.com/site/about/guidelines.xhtml

Table 2 Multivariable logistic regression models predicting desire to weigh less and attempting to lose weight in a sample of overweight and obese ~~UK~~ British adults (n=810)~~(weighted data)~~

	Desire to weigh less			Attempting to lose weight		
**Characteristics**	**OR**	**95% CI**	***P***	**OR**	**95% CI**	***P***
Age (years)						
<55	1.00	-	-	1.00	-	-
≥55	0.84	0.57-1.23	0.369	0.52	0.38-0.71	<0.001
Sex						
Male	1.00	-	-	1.00	-	-
Female	4.39	2.94-6.56	<0.001	1.93	1.43-2.63	<0.001
Ethnicity						
White	1.00	-	-	1.00	-	-
Non-white	0.96	0.51-1.84	0.912	1.11	0.67-1.85	0.686
Marital status						
Unmarried	1.00	-	-	1.00	-	-
Married/living as married	0.87	0.59-1.29	0.483	1.34	0.98-1.84	0.065
SES						
Lower	1.00	-	-	1.00	-	-
Higher	1.34	0.92-1.95	0.125	1.40	1.03-1.89	0.030
Weight status						
Overweight	1.00	-	-	1.00	-	-
Obese	8.57	4.87-15.08	<0.001	1.91	1.38-2.64	<0.001
Health professional advice to lose weight						
No	1.00	-	-	1.00	-	-
Yes	3.71	2.10-6.55	<0.001	3.53	2.44-5.10	<0.001

Data were weighted on age, sex, social grade, and standard region. Results were not notably different when analyses were run on unweighted data.
All variables entered into the models are shown in the table; there were no additional covariates.
SES, ~~=~~ socio~~-~~economic status; OR, ~~=~~ odds ratio; CI, ~~=~~ confidence interval.

For peer review only

For peer review only - http://bmjopen.bmj.com/site/about/guidelines.xhtml

FIGURES

Figure 1 Percentage of overweight and obese British adults reporting ever having received health professional advice to lose weight by BMI

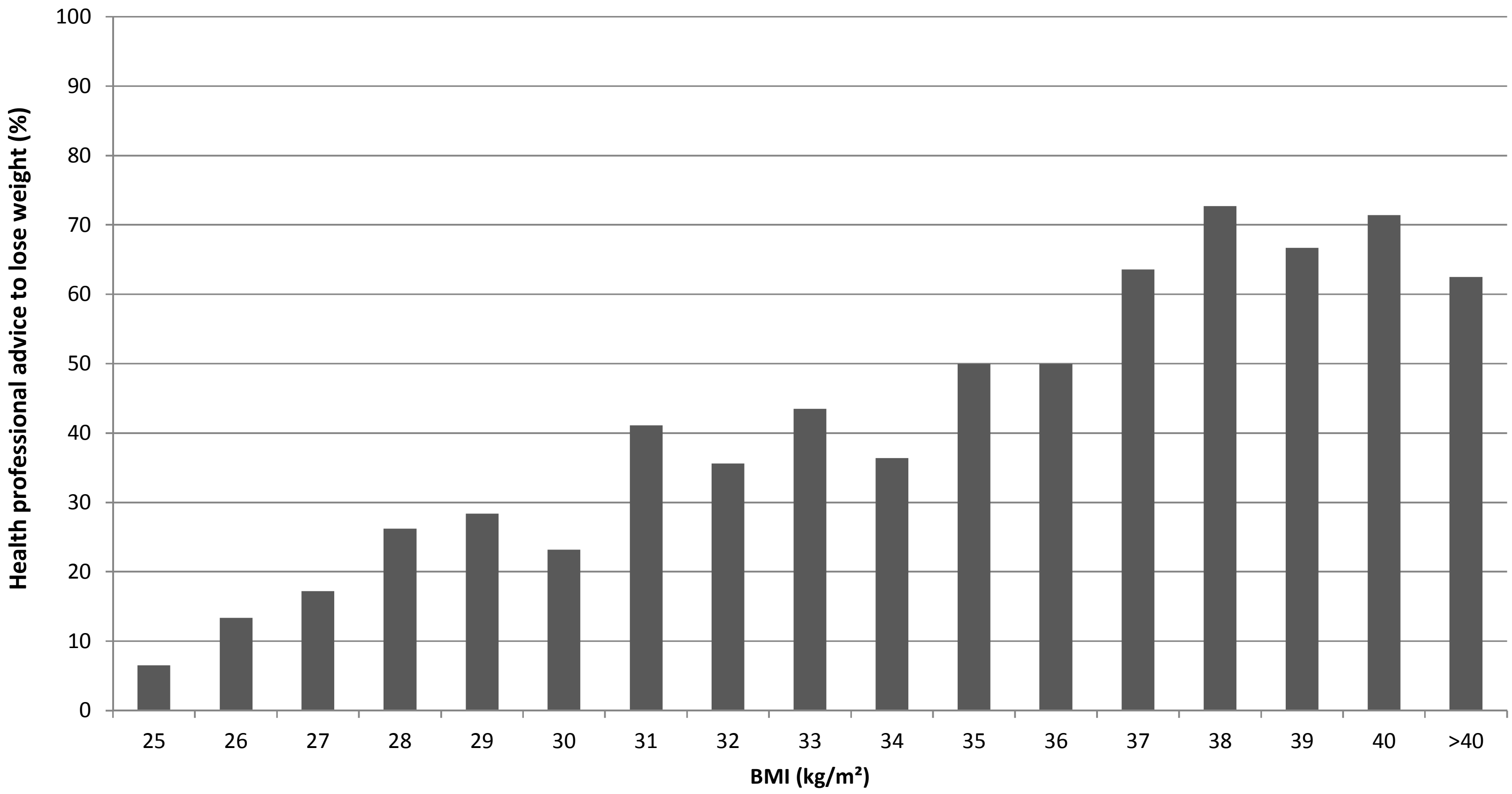

~~Note:~~Unweighted data shown.
BMI points were rounded down such that a BMI point of 25 includes 25.0-25.9.

Figure 2 Prevalence of attempting to lose weight in overweight and obese British adults by health professional advice to lose weight (yes/no) and weight status (overweight/obese)

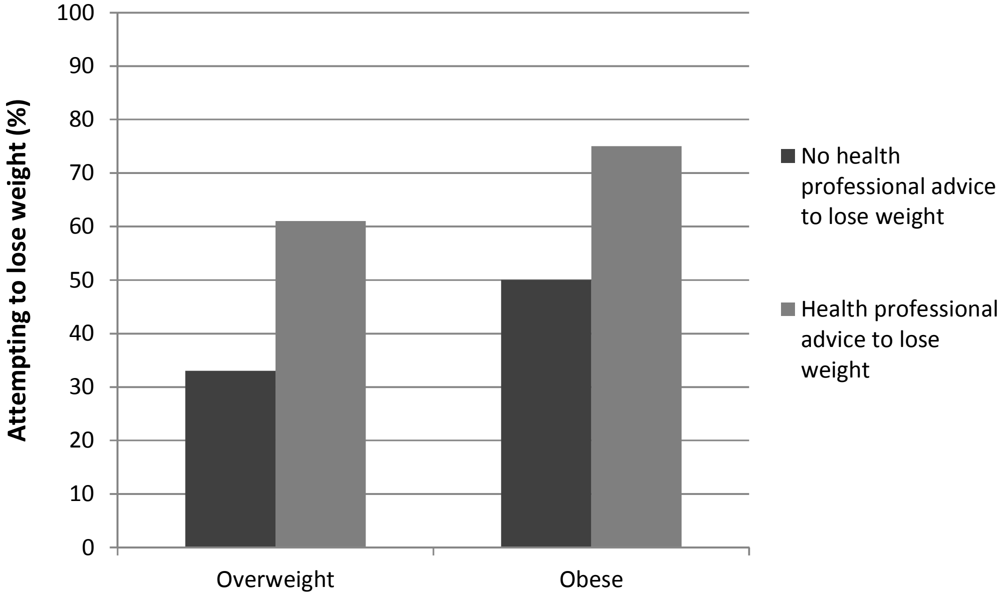

Unweighted data shown.

For peer review only - http://bmjopen.bmj.com/site/about/guidelines.xhtml

CONTRIBUTORSHIP STATEMENT

Everyone listed as an author fulfils all three of the ICMJE guidelines for authorship: 1) substantial contributions to conception and design, acquisition of data, or analysis and interpretation of data; 2) drafting the article or revising it critically for important intellectual content; and 3) final approval of the version to be published.

S~~E~~J, JW, FJ, NF, and R~~J~~B were responsible for the study concept and design. JW obtained funding. JW and FJ acquired the data. S~~E~~J, JW, FJ, NF, and R~~J~~B analysed and interpreted the data. S~~E~~J did the statistical analysis. S~~E~~J drafted the manuscript, and all authors revised it for important intellectual content. All authors had final approval of the version to be published. JW is the guarantor.

All authors had full access to all of the data (including statistical reports and tables) in the study and can take responsibility for the integrity of the data and the accuracy of the data analysis.

COMPETING INTERESTS

All authors have completed the Unified Competing Interest form at www.icmje.org/coi_disclosure.pdf (available on request from the corresponding author) and declare: support for the study by grants from the UK Medical Research Council, UK Economic and Social Research Council, and Cancer Research UK; no financial relationships with any organisations that might have an interest in the submitted work in the previous three years; and no other relationships or activities that could appear to have influenced the submitted work.

FUNDING

This study was supported by grants from the UK Medical Research Council, UK Economic and Social Research Council, and Cancer Research UK.

The study sponsors had no role in the study design; in the collection, analysis, and interpretation of the data; in the preparation, review, or approval of the manuscript; or in the decision to submit it for publication. The researchers were independent of the funding agencies.

For peer review only

For peer review only - http://bmjopen.bmj.com/site/about/guidelines.xhtml

REFERENCES

1. Guh DP, Zhang W, Bansback N, Amarsi Z, Laird Birmingham C, Anis AH. The incidence of co-morbidities related to obesity and overweight: A systematic review and meta-analysis. BMC Public Health 2009;**9**:88 (2009).
2. Ford ES, Mokdad AH. Epidemiology of obesity in the western hemisphere. JCEM 2008;**93**:S1–S8.
3. Blackburn, G. Effect of degree of weight loss on health benefits. Obes Res 1995;**3**:S211–S216.
4. Lavie CJ, Milani RV, Artham SM, Patel D, Ventura HO. The obesity paradox, weight loss, and coronary disease. Am J Med 2009;**122**:1106–1114.
5. Franz MJ, VanWormer JJ, Crain AL, Boucher JL, Histon T, Caplan W et al. Weight-loss outcomes: a systematic review and meta-analysis of weight-loss clinical trials with a minimum 1-year follow-up. J Am Diet Ass 2007;**107**:1755–1767.
6. Tsai AG, Wadden TA. Systematic review: an evaluation of major commercial weight loss programs in the United States. Ann Intern Med 2005;**142**:56–66.
7. Yaemsiri S, Slining MM, Agarwal SK. Perceived weight status, overweight diagnosis, and weight control among US adults: the NHANES 2003–2008 Study. Int J Obes 2011;**35**:1063–1070.
8. Wardle J, Johnson F. Weight and dieting: examining levels of weight concern in British adults. Int J Obes 2002;**26**:1144–1149.
9. Moyer VA. Screening for and management of obesity in adults: U.S. Preventive Services Task Force recommendation statement. Ann Intern Med 2012;**157**:373–378 (2012).
10. Academy of Medical Royal Colleges. Measuring Up: The medical profession's prescription for the nation's obesity crisis. Academy of Medical Royal Colleges, 2013. http://www.aomrc.org.uk/projects/obesity-steering-group.html (accessed 17 Jun 2013).
11. Royal College of Physicians. The training of health professionals for the prevention and treatment of overweight and obesity. Royal College of Physicians, 2010. http://www.rcplondon.ac.uk/sites/default/files/press_releases/2010/12/obesity-report-2010.pdf (accessed 17 Jun 2013).

~~12. NHS Employers. 2013/14 general medical services (GMS) contract quality and outcomes framework (QOF). 2013. http://www.nhsemployers.org/Aboutus/Publications/Documents/qof-2013-14.pdf (accessed 17 Jun 2013).~~

~~13~~12. National Institute for Health and Clinical Excellence (NICE). Obesity: the prevention, identification, assessment and management of overweight and obesity in adults and children. NICE, 2006. http://www.nice.org.uk/ (accessed 17 Jun 2013).

~~14. Scottish Intercollegiate Guidelines Network (SIGN). Management of Obesity: A national clinical guideline. SIGN, 2010. http://www.sign.ac.uk/guidelines/fulltext/115/ (accessed 17 Jun 2013).~~

~~15~~13. Clune A, Fischer JG, Lee JS, Reddy S, Johnson MA, Hausman DB. Prevalence and predictors of recommendations to lose weight in overweight and obese older adults in Georgia senior centers. Prev Med 2010;**51**:27–30.

For peer review only - http://bmjopen.bmj.com/site/about/guidelines.xhtml

~~16~~14. Galuska DA, Will JC, Serdula MK, Ford ES. Are health care professionals advising obese patients to lose weight? JAMA 1999;**282**:1576–1578.

~~17~~15. Jackson JE, Doescher MP, Saver BG, Hart LG. Trends in professional advice to lose weight among obese adults, 1994 to 2000. J Gen Intern Med 2005;**20**:814–818.

~~18~~16. Loureiro ML, Nayga Jr RM. Obesity, weight loss, and physician's advice. Soc Sci Med 2006;**62**:2458–2468.

~~19~~17. Sciamanna C, Tate D, Lang W, Wing RR. Who reports receiving advice to lose weight?: Results from a multistate survey. Arch Intern Med 2000;**160**:2334–2339.

~~20~~18. Stafford RS, Farhat JH, Misra B, Schoenfeld DA. National patterns of physician activities related to obesity management. Arch Fam Med 2000;**9**:631–638.

~~21~~19. Hiddink GJ, Hautvast JG, Van Woerkum CM, Fieren CJ, Van 't Hof MA. Nutrition guidance by primary-care physicians: perceived barriers and low involvement. Eur J Clin Nutr 1995;**49**:842–851.

~~22~~20. Harris JE, Hamaday V, Mochan E. Osteopathic family physicians' attitudes, knowledge, and self-reported practices regarding obesity. J Am Osteopath Assoc 1999;**99**:358–365.

~~23~~21. Ruelaz AR, Diefenbach P, Simon B, Lanto A, Arterburn D, Shekelle PG. Perceived barriers to weight management in primary care--perspectives of patients and providers. J Gen Intern Med 2007;**22**:518–522.

~~24~~22. Kenner MM, Taylor ML, Dunn PC, Gruchow HW, Kolasa K. primary care providers need a variety of nutrition and wellness patient education materials. J Am Diet Assoc 1999;**99**:462–466.

~~25~~23. Leverence RR, Williams RL, Sussman A, Crabtree BF. Obesity counseling and guidelines in primary care: a qualitative study. Am J Prev Med 2007;**32**:334–339.

~~26~~24. Foster GD, Wadden TA, Makris AP, Davidson D, Sanderson RS, Allison DB et al. Primary care physicians' attitudes about obesity and its treatment. Obes Res 2003;**11**:1168–1177.

~~27~~25. Michie S. Talking to primary care patients about weight: A study of GPs and practice nurses in the UK. Psychol Health Med 2007;**12**:521–525.

~~28~~26. Frank A. Futility and avoidance. Medical professionals in the treatment of obesity. JAMA 1993;**269**:2132–2133.

~~29~~27. West R, McNeill A, Raw M. Smoking cessation guidelines for health professionals: an update. Thorax 2000;**55**:987–999.

~~30~~28. Rose SA, Poynter PS, Anderson JW, Noar SM, Conigliaro J. Physician weight loss advice and patient weight loss behavior change: a literature review and meta-analysis of survey data. Int J Obes 2013;**37**:118-128.

~~31~~29. Johnson F, Cooke L, Croker H, Wardle J. Changing perceptions of weight in Great Britain: comparison of two population surveys. BMJ 2008;**337**:a494–a494.

~~32~~30. National Readership Survey. Social grade - definitions and discriminatory power. National Readership Survey, 2007. http://www.nrs.co.uk/lifestyle.html (accessed 17 Jun 2013).

31. Bardia A, Holtan SG, Slezak JM, Thompson WG. Diagnosis of obesity by primary care physicians and impact on obesity management. Mayo Clin Proc 2007;**82**:927–932.

32. Huang J, Yu H, Marin E, Brock S, Carden D, Davis T. Physicians' weight loss counseling in two public hospital primary care clinics. Acad Med 2004;**79**:156–161.

33. O'Brien SH, Holubkov R, Reis EC. Identification, evaluation, and management of obesity in an academic primary care center. Pediatrics 2004;**114**:e154–e159.

For peer review only

For peer review only - http://bmjopen.bmj.com/site/about/guidelines.xhtml

~~33~~34. Bocquier A, Verger P, Basdevant A, Andreotti G, Baretge J, Villani P et al. Overweight and obesity: knowledge, attitudes, and practices of general practitioners in France. Obes Res 2005;**13**:787–795.

~~34~~35. Campbell K, Crawford D. Management of obesity: attitudes and practices of Australian dietitians. Int J Obes Relat Metab Disord 2000;**24**:701–710.

~~35~~36. Campbell K, Engel H, Timperio A, Cooper C, Crawford D. Obesity management: Australian general practitioners' attitudes and practices. Obes Res 2000;**8**:459–466.

~~36~~37. Loomis GA, Connolly KP, Clinch CR, Djuric DA. Attitudes and practices of military family physicians regarding obesity. Mil Med 2001;**166**:121–125.

~~37~~38. Thuan JF, Avignon A. Obesity management: attitudes and practices of French general practitioners in a region of France. Int J Obes 2005;**29**:1100–1106.

~~38~~39. Webb TL, Sheeran P. Does changing behavioral intentions engender behavior change? A meta-analysis of the experimental evidence. Psychol Bull 2006;**132**:249–268.

~~39~~40. Cameron R, Evers SE. Self-report issues in obesity and weight management: State of the art and future directions. Behav Assess 1990. http://psycnet.apa.org/psycinfo/1990-18712-001 (accessed 17 Jun 2013).

~~40~~41. Roberts RJ. Can self-reported data accurately describe the prevalence of overweight? Public Health 1995;**109**:275–284.

~~41~~42. Health Survey for England. Health Survey for England - 2010: Trend tables. Health Survey for England, 2010. http://www.ic.nhs.uk/statistics-and-data-collections/health-and-lifestyles-related-surveys/health-survey-for-england/health-survey-for-england--2010-trend-tables (accessed 17 Jun 2013).

~~42~~43. Laws R. Current approaches to obesity management in UK Primary Care: the Counterweight Programme. J Hum Nutr Diet 2004;**17**:183–190.

44. Franz MJ, VanWormer JJ, Crain AL, Boucher JL, Histon T, Caplan W et al. Weight-loss outcomes: a systematic review and meta-analysis of weight-loss clinical trials with a minimum 1-year follow-up. J Am Diet Ass 2007;**107**:1755–1767.

For peer review only

For peer review only - http://bmjopen.bmj.com/site/about/guidelines.xhtml

STROBE Statement—checklist of items that should be included in reports of observational studies

	Item No	Recommendation	
**Title and abstract**	1	(*a*) Indicate the study's design with a commonly used term in the title or the abstract	Yes
		(*b*) Provide in the abstract an informative and balanced summary of what was done and what was found	Yes
**Introduction**			
Background/rationale	2	Explain the scientific background and rationale for the investigation being reported	Yes
Objectives	3	State specific objectives, including any prespecified hypotheses	Yes
**Methods**			
Study design	4	Present key elements of study design early in the paper	Yes
Setting	5	Describe the setting, locations, and relevant dates, including periods of recruitment, exposure, follow-up, and data collection	Yes
Participants	6	(*a*) *Cohort study*—Give the eligibility criteria, and the sources and methods of selection of participants. Describe methods of follow-up<br>*Case-control study*—Give the eligibility criteria, and the sources and methods of case ascertainment and control selection. Give the rationale for the choice of cases and controls<br>*Cross-sectional study*—Give the eligibility criteria, and the sources and methods of selection of participants	Yes
		(*b*) *Cohort study*—For matched studies, give matching criteria and number of exposed and unexposed<br>*Case-control study*—For matched studies, give matching criteria and the number of controls per case	NA
Variables	7	Clearly define all outcomes, exposures, predictors, potential confounders, and effect modifiers. Give diagnostic criteria, if applicable	Yes
Data sources/ measurement	8*	For each variable of interest, give sources of data and details of methods of assessment (measurement). Describe comparability of assessment methods if there is more than one group	Yes
Bias	9	Describe any efforts to address potential sources of bias	Yes
Study size	10	Explain how the study size was arrived at	Yes
Quantitative variables	11	Explain how quantitative variables were handled in the analyses. If applicable, describe which groupings were chosen and why	Yes
Statistical methods	12	(*a*) Describe all statistical methods, including those used to control for confounding	Yes
		(*b*) Describe any methods used to examine subgroups and interactions	NA
		(*c*) Explain how missing data were addressed	Yes
		(*d*) *Cohort study*—If applicable, explain how loss to follow-up was addressed<br>*Case-control study*—If applicable, explain how matching of cases and controls was addressed<br>*Cross-sectional study*—If applicable, describe analytical methods taking account of sampling strategy	Yes
		(*e*) Describe any sensitivity analyses	NA

Continued on next page

For peer review only

**Results**			
Participants	13*	(a) Report numbers of individuals at each stage of study—eg numbers potentially eligible, examined for eligibility, confirmed eligible, included in the study, completing follow-up, and analysed	Yes
		(b) Give reasons for non-participation at each stage	Yes
		(c) Consider use of a flow diagram	No
Descriptive data	14*	(a) Give characteristics of study participants (eg demographic, clinical, social) and information on exposures and potential confounders	Yes
		(b) Indicate number of participants with missing data for each variable of interest	Yes
		(c) *Cohort study*—Summarise follow-up time (eg, average and total amount)	NA
Outcome data	15*	*Cohort study*—Report numbers of outcome events or summary measures over time	NA
		Case-control study—Report numbers in each exposure category, or summary measures of exposure	NA
		Cross-sectional study—Report numbers of outcome events or summary measures	Yes
Main results	16	(*a*) Give unadjusted estimates and, if applicable, confounder-adjusted estimates and their precision (eg, 95% confidence interval). Make clear which confounders were adjusted for and why they were included	Yes
		(*b*) Report category boundaries when continuous variables were categorized	NA
		(*c*) If relevant, consider translating estimates of relative risk into absolute risk for a meaningful time period	NA
Other analyses	17	Report other analyses done—eg analyses of subgroups and interactions, and sensitivity analyses	NA
**Discussion**			
Key results	18	Summarise key results with reference to study objectives	Yes
Limitations	19	Discuss limitations of the study, taking into account sources of potential bias or imprecision. Discuss both direction and magnitude of any potential bias	Yes
Interpretation	20	Give a cautious overall interpretation of results considering objectives, limitations, multiplicity of analyses, results from similar studies, and other relevant evidence	Yes
Generalisability	21	Discuss the generalisability (external validity) of the study results	Yes
**Other information**			
Funding	22	Give the source of funding and the role of the funders for the present study and, if applicable, for the original study on which the present article is based	Yes

*Give information separately for cases and controls in case-control studies and, if applicable, for exposed and unexposed groups in cohort and cross-sectional studies.

Note: An Explanation and Elaboration article discusses each checklist item and gives methodological background and published examples of transparent reporting. The STROBE checklist is best used in conjunction with this article (freely available on the Web sites of PLoS Medicine at http://www.plosmedicine.org/, Annals of Internal Medicine at http://www.annals.org/, and Epidemiology at http://www.epidem.com/). Information on the STROBE Initiative is available at www.strobe-statement.org.

For peer review only
